# Plasmon-induced selective carbon dioxide conversion on earth-abundant aluminum-cuprous oxide antenna-reactor nanoparticles

**DOI:** 10.1038/s41467-017-00055-z

**Published:** 2017-06-21

**Authors:** Hossein Robatjazi, Hangqi Zhao, Dayne F. Swearer, Nathaniel J. Hogan, Linan Zhou, Alessandro Alabastri, Michael J. McClain, Peter Nordlander, Naomi J. Halas

**Affiliations:** 1 Department of Electrical and Computer Engineering, Rice University, Houston, TX 77005 USA; 2 Laboratory for Nanophotonics, Rice University, Houston, TX 77005 USA; 3 Department of Chemistry, Rice University, Houston, TX 77005 USA; 4 Department of Physics and Astronomy, Rice University, Houston, TX 77005 USA; 5 Department of Material Science and Nanoengineering, Rice University, Houston, TX 77005 USA

## Abstract

The rational combination of plasmonic nanoantennas with active transition metal-based catalysts, known as ‘antenna-reactor’ nanostructures, holds promise to expand the scope of chemical reactions possible with plasmonic photocatalysis. Here, we report earth-abundant embedded aluminum in cuprous oxide antenna-reactor heterostructures that operate more effectively and selectively for the reverse water-gas shift reaction under milder illumination than in conventional thermal conditions. Through rigorous comparison of the spatial temperature profile, optical absorption, and integrated electric field enhancement of the catalyst, we have been able to distinguish between competing photothermal and hot-carrier driven mechanistic pathways. The antenna-reactor geometry efficiently harnesses the plasmon resonance of aluminum to supply energetic hot-carriers and increases optical absorption in cuprous oxide for selective carbon dioxide conversion to carbon monoxide with visible light. The transition from noble metals to aluminum based antenna-reactor heterostructures in plasmonic photocatalysis provides a sustainable route to high-value chemicals and reaffirms the practical potential of plasmon-mediated chemical transformations.

## Abstract (web summary)

Plasmon-enhanced photocatalysis holds promise for the control of chemical reactions. Here the authors report an Al@Cu_2_O heterostructure based on earth abundant materials to transform CO_2_ into CO at significantly milder conditions.

## Introduction

Heterogeneous catalysis is at the heart of the chemical industry^1^. Each year, trillions of dollars of value-added chemicals are manufactured using heterogeneous catalytic processes that rely on active transition metal catalysts such as Pd, Ru, Rh, Cu or Ni^2, 3^. Unfortunately, transition metal catalysts frequently demand energy-intensive conditions, such as high pressure and heat, to shift equilibrium toward the formation of products. To minimize energy consumption and cost, while achieving maximum efficiency in large-scale chemical processes, new alternatives to extreme operating conditions need to be pursued. This challenge can be addressed by introducing robust, light-activated photocatalysts that lower reaction barriers at ambient conditions instead of relying on traditional, phonon-driven chemical conversion.

Recent advances have brought metal nanoparticles that support localized surface plasmon resonances (LSPR) to the forefront for enhancing photocatalytic efficiencies^4–13^. Until recently, light-driven chemical transformation on plasmonic nanostructures has relied primarily on Ag and Au. However, practical applications of noble metals as photocatalysts are ultimately limited due to their high cost, low abundance, and modest reactivity. In contrast, aluminum (Al), the most abundant metal in the Earth’s crust, also has outstanding plasmonic optical properties, including a highly size-tunable localized plasmon resonance that spans the UV and visible regions of the optical spectrum^14^. Al has been demonstrated to be a photocatalyst for plasmon-mediated chemical reactions^15^ and has recently been adopted as a plasmonic antenna in a heterometallic antenna-reactor photocatalyst design^16, 17^. Taking into account its low cost, abundance, and plasmonic and catalytic activity, Al is a highly promising candidate for eventual industrial implementation of plasmonic photocatalysis.

Here we demonstrate a plasmonic Al@Cu_2_O antenna-reactor heterostructure based on entirely earth-abundant materials instead of precious metals for charge-carrier generation to drive chemical transformation of CO_2_ into CO at significantly milder operating conditions compared to its purely thermal driven counterpart. The conversion of CO_2_ to CO is considered to be an important approach for the mitigation of anthropogenic CO_2_. Currently, industrial conversion of CO_2_ to CO occurs through the reverse water-gas shift (rWGS) reaction. The rWGS (CO_2_+H_2_→CO+H_2_O ΔH_298 K_ = +41.2 kJ mol^−1^) has emerged not only as a promising approach for CO_2_ transformation into CO but also as an important reactant for synthesis of high value chemicals by subsequent hydrogenation (i.e., the Fisher–Tropsch synthesis)^18^. We observe better selectivity in the plasmon-induced rWGS at lower temperatures than the thermally driven rWGS at elevated temperatures. Through combined experimental studies and theoretical modeling of the Al@Cu_2_O photocatalyst using finite element method (FEM) electromagnetic and Monte-Carlo simulations, we are also able to provide definitive evidence to support plasmon-induced carrier generation mechanism to drive rWGS, and to clearly distinguish it from photothermal heating. Our results serve as an example of Al as a plasmonic antenna promoting reactivity on adjacent materials, in this case, the semiconducting oxide Cu_2_O. The antenna-reactor geometry not only improves surface reactivity, but also more efficiently harnesses and utilizes the radiative LSPR damping in Al to enhance carrier generation in the metal oxide shell. Rational design and independent control over the catalytic and light-harvesting components in the Al@Cu_2_O heterostructure results in an efficient and selective plasmonic photocatalyst.

## Results

### Photocatalyst characterization

**Fig. 1:**
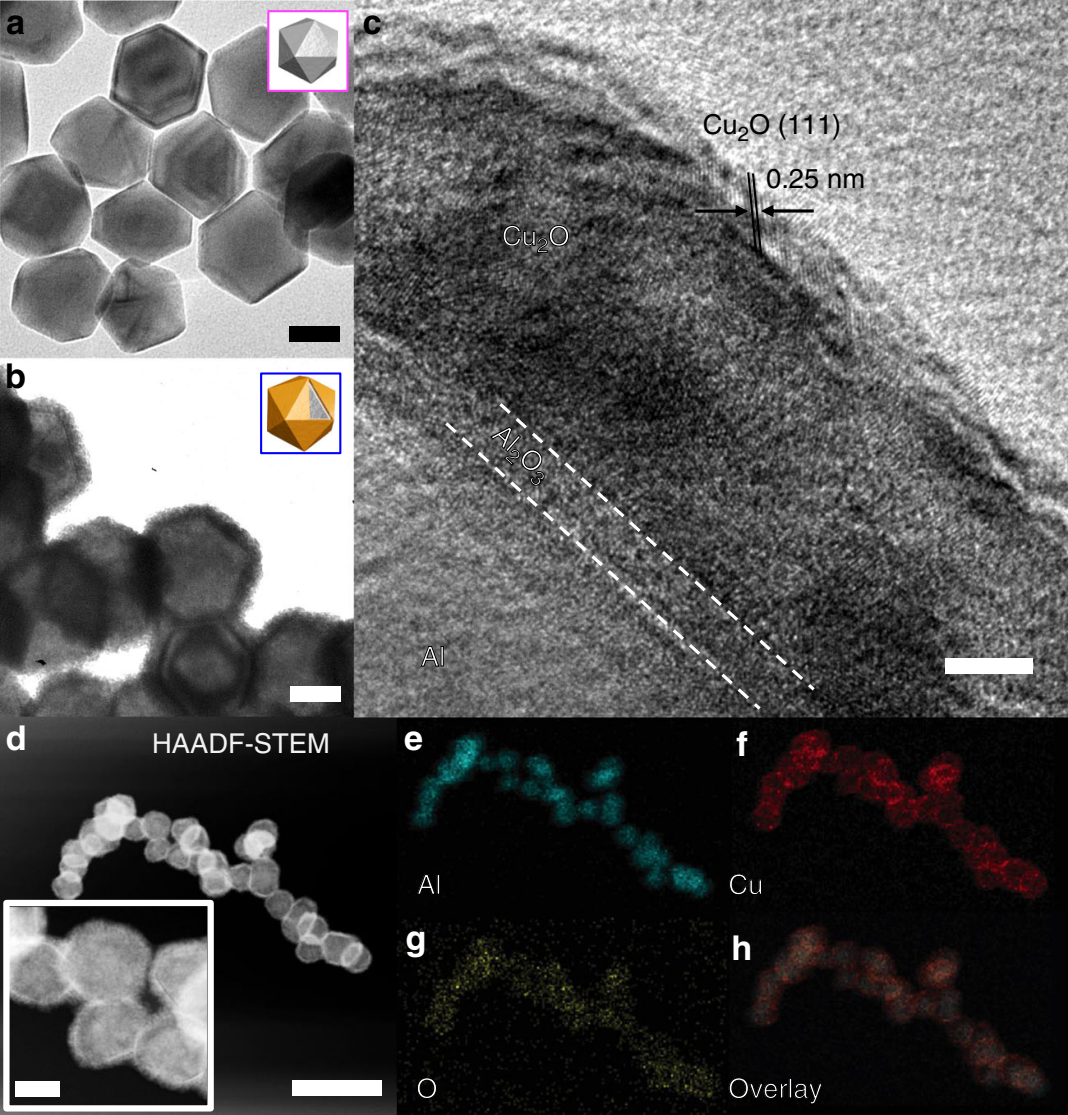
Characterization of plasmonic photocatalysts. **a** TEM image of the pristine Al NCs with nominal size before and **b** after growth of Cu_2_O shell around Al core. The scale bars in **a** and **b** are 50 nm. **c** HRTEM image of Al/Al_2_O_3_/Cu_2_O showing the Cu_2_O layer is highly polycrystalline. The scale bar is 5 nm. **d** High-angle annular dark field scanning transmission electron micrograph (HAADF-STEM) of the Al@Cu_2_O particles in low magnification and higher resolution (inset) indicating different Z-contrast for the core and the shell materials. The scale bars for low-resolution and inset images are 400, and 50 nm, respectively. **e**–**h** Energy-dispersive X-ray (EDX) mapping showing the distribution of Al **e**, Cu **f** and O **g**, and their overlay **h**

Electron microscopy and elemental composition analysis of the particles are shown in Fig. 1. The pristine Al nanocrystals (Al NCs) with a nominal diameter of 100 nm (Fig. 1a; Supplementary Fig. 1) were colloidally synthesized according to our previously published protocol^19^ with minor modification. To prepare the plasmonic antenna-reactor nanoparticles, we grew a ~15 nm thick cuprous oxide (Cu_2_O) shell around the Al core (Figs. 1b and c; Supplementary Fig. 1), which was separated from the Al metal by a 2–4 nm self-limiting amorphous Al_2_O_3_ layer (see Methods for synthesis details). A high-resolution transmission electron micrograph (HRTEM) of the as-synthesized Al@Cu_2_O nanoparticles revealed that the shell was highly polycrystalline, with lattice fringes that can be assigned to the (111) (Fig. 1c) and (110) (Supplementary Fig. 1) planes of Cu_2_O. The high-angle annular dark field scanning transmission electron micrograph (HAADF-STEM) of particles in Fig. 1d shows a strong contrast between bright and dark regions, which arises from the Z-contrast of different atomic nuclei: Cu atoms appear brighter than Al due to their higher atomic number. The elemental compositions were mapped using energy dispersive x-ray spectroscopy. The elemental mapping (Figs. 1e–h) illustrates the distribution of each element in the nanoparticles. The HAADF-STEM image and combined elemental mapping shows that coating the Al NCs surface with Cu_2_O shell is achieved in high quality. The UV–vis-NIR extinction spectrum (Supplementary Fig. 2) in solution shows a dipolar plasmon mode at 465 nm for pristine Al NCs that shifts to ~550 nm after growth of the Cu_2_O shell. Diffuse reflectance spectra of the photocatalyst nanoparticle/γ-Al_2_O_3_ mixture were obtained, where both indicated the strong interband transition of Al (Supplementary Fig. 3). The X-ray photoelectron spectroscopy analysis shows identical spectrum at binding energies of the copper in the Al@Cu_2_O/γ-Al_2_O_3_ photocatalyst before and following illumination under reaction conditions, confirming the oxidation state of copper in the Al@Cu_2_O nanoparticles remained unchanged during photocatalytic reaction (Supplementary Fig. 4 and Supplementary Method 2).

### Plasmon-enhanced rWGS

**Fig. 2:**
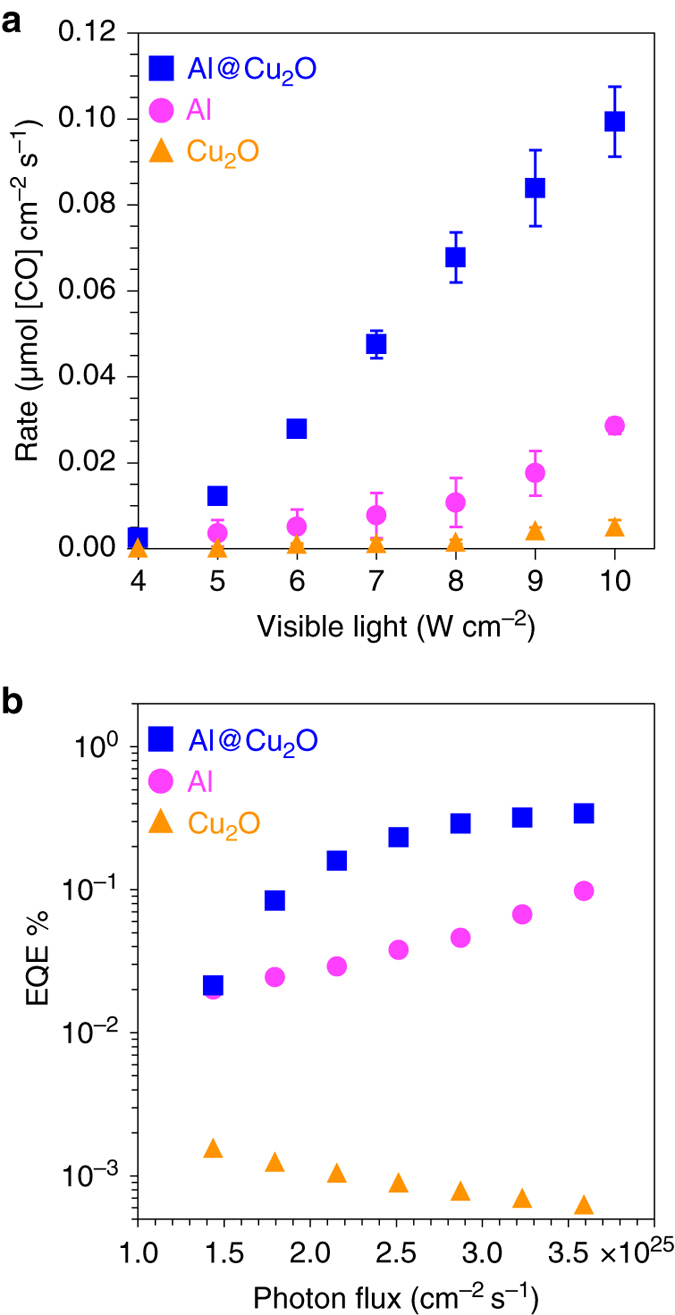
Plasmon-enhanced rWGS. **a** The impact of visible light intensity on the rate of CO formation on photocatalysts prepared from Cu_2_O, Al NCs and Al@Cu_2_O. **b** The apparent external quantum efficiency (EQE) calculated from measured reaction rate in **a** and plotted against photon flux

The photocatalysts used in this study were prepared from a homogeneous dispersion of plasmonic nanoparticles dispersed into a high surface area γ-Al_2_O_3_ support at 5 wt%. Photocatalytic measurements were performed using about 20 mg of nanoparticle/γ-Al_2_O_3_ mixture, loaded into a fixed-bed reactor equipped with a quartz window. The chamber outlet was connected to a gas chromatograph for on-line quantitative analysis of products in effluent (see Methods section for details). Preliminary photocatalytic CO_2_ hydrogenation experiments were performed in CO_2_ and H_2_ (1:1, total flow rate of 10 s.c.c.m.) and also separately in He (10 s.c.c.m.) atmospheres under visible light illumination (Supplementary Fig. 5). CO formation was detected when CO_2_ and H_2_ were both present, upon illumination of the nanoparticle/γ-Al_2_O_3_ photocatalyst mixture. Illumination of the photocatalyst in an inert He atmosphere did not produce any measurable product, verifying CO formation was not from the decomposition of any organic contamination. Also, there was no measurable product from illuminating the pure γ-Al_2_O_3_, verifying that the Al@Cu_2_O plasmonic photocatalyst was the active component. The rate of CO formation as a function of visible-light intensity under ambient conditions is shown in Fig. 2a. The rate of CO formation catalyzed by Al@Cu_2_O is significantly higher than that of Cu_2_O and pristine Al without the reactive Cu_2_O shell, particularly at higher illumination intensities. Similarly, the Al@Cu_2_O heterostructure exhibits higher external quantum efficiencies (EQE) of CO formation (Fig. 2b). The positive dependence of EQE on incident photon flux is a distinct feature of plasmon-induced charge-carrier driven photocatalysis (reaction rate *α* intensity, where *n* > 1)^20^. On the contrary, increasing irradiation intensities on semiconductor surfaces does not improve EQE as verified by pure Cu_2_O shown in Fig. 2b ^21^.

Figure 2 shows an average CO formation rate of about 360 µmole cm^–2^ h^–1^ on oxide supported Al@Cu_2_O nanoparticles at an incident photon flux of 10 W cm^–2^. The measured rate of CO formation is on par with or higher than previous reports on photocatalytic CO_2_ conversion^22^. We also note that the measured EQE of about 0.35% for Al@Cu_2_O is higher than previous reports for plasmon-enhanced CO_2_ conversion over Au and Ag^23–25^. However, significant diversity in materials and experimental conditions make direct comparison of reported efficiencies for photocatalytic CO_2_ conversion a challenge. The majority of homogenous and heterogeneous photocatalytic systems for CO_2_ conversion have relied on rare elements. They often suffer from very low aqueous CO_2_ solubility, utilize expensive co-catalysts and/or sacrificial organics, and in many cases, utilize an electrochemical bias or elevated temperatures to activate the photocatalyst. Despite a measured quantum efficiency of less than 1%, plasmon-induced rWGS at room temperature and pressure, all while utilizing a non-precious, earth-abundant Al@Cu_2_O antenna-reactor photocatalyst holds promise for light-driven CO_2_ mitigation.

### Plasmonic carrier-driven mechanism and photothermal effects

In principle, plasmon-induced chemistry should always be accompanied by heat generation from the thermalization of the hot carriers generated by plasmon decay, through the mechanism of nonradiative plasmon damping^8^. Due to the short lifetime (fs) of the hot carriers compared to chemical reaction timescales (ps), only a portion of the plasmonically generated hot carriers will contribute to the photocatalytic activity. All remaining carriers eventually undergo energy relaxation through heat dissipation into the crystal lattice and environment. While previous studies^5, 7, 20^ have clearly provided evidence for a hot electron-driven mechanism for photocatalysis, it is still critical to investigate the relative contributions of nonthermal and photothermal contributions in any given photocatalytic reaction. In this regard, we performed a series of experimental studies supported by theoretical models to differentiate between the contributions of plasmon-induced carrier generation and photothermal heating in the case of the photocatalytic rWGS reaction on Al@Cu_2_O. We observe that despite a temperature increase due to plasmonic photothermal heating of the photocatalyst, photogenerated carriers rather than photothermal heating drive CO formation.

**Fig. 3:**
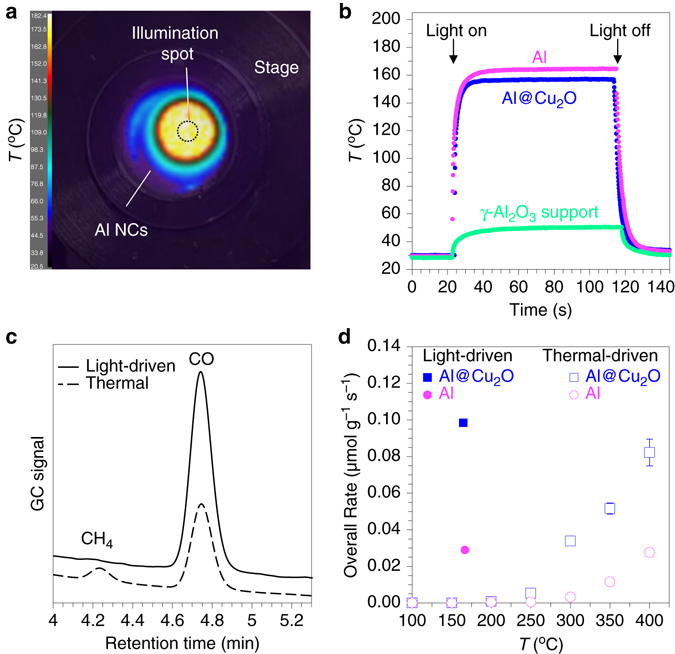
Light-driven vs. thermal-driven activity characterization for rWGS. **a** Spatial temperature mapping of the catalysts surface during illumination of Al NCs/γ-Al_2_O_3_ in air under visible light intensity of 10 W cm^−2^. **b** Steady-state temperature monitoring for oxide supported plasmonic nanoparticles compared to pure oxide support with and without irradiation in air. **c** Typical gas chromatogram of the chamber output during light (7 W cm^−2^) and thermal driven (350 °C) rWGS on Al@Cu_2_O. **d** The overall rate of products formation as a function of applied temperature in purely thermal-driven (light off) rWGS for oxide supported Al NCs and Al@Cu_2_O (unfilled data points). For comparison, the reaction rates during the light-induced process (10 W cm^−2^) are shown at the corresponding recorded temperatures for oxide supported Al NCs and Al@Cu_2_O (filled data points)

Initially, the maximum local temperature on the surface of the single Al@Cu_2_O was calculated as a function of illumination intensity and wavelength (Supplementary Fig. 6 and Supplementary Note 1), which resulted in negligible temperature increase under illumination conditions. The steady-state measurement of surface temperature using in-situ Raman spectroscopy was recently demonstrated during photocatalytic rWGS on low density dispersion of Au NPs in ZnO matrix; surface temperatures in the range of about 30 °C and 600 °C was measured corresponding to laser intensities in the range of 12 W cm^–2^ and 80 W cm^–2^ for illumination at plasmon resonance of Au^23^. Here, we investigated the steady-state heating of our photocatalysts under illumination using high-resolution spatial and temporal mapping of temperature variations during irradiation (Figs. 3a and b; Supplementary Fig. 7). At our maximum visible light intensity of 10 W cm^–2^ in air, the Al NCs/γ-Al_2_O_3_ catalyst surface temperature reached upwards of 175 °C as shown in the temperature profile map of the catalysts surface in Fig. 3a. This temperature increase near the illuminated surface is significantly higher than previously reported under similar illumination intensity^23^ and our calculated local temperature increase for single particle surface.

The difference can be attributed to the collective heating effect mediated by multiple light scattering and absorption events by randomly dispersed particles under illumination volume^26^. Consequently, the heated area in Fig. 3a is much larger than the incident beam size. As shown in the steady-state temperature variation profile (Fig. 3b), upon loading (5 wt%) of plasmonic nanoparticles into the pure oxide support, the average surface temperature under illumination (10 W cm^–2^) increases significantly (Δ*T*~110 °C). This additional temperature increase corresponds to photothermal heating from electron–electron scattering in the hot-carrier decay pathway. However, despite temperature increases due to plasmonic photothermal heating, we will show here that the photothermal effect does not play the major role in photocatalytic rWGS.

An analysis of the reaction products during light-induced and purely thermally driven (light off) rWGS (Fig. 3c) revealed that in contrast to the highly selective CO formation during photocatalytic rWGS, the thermally driven process results in the formation of CH_4_ and CO with selectivity of CO/CH_4_ varying between 50 and 97% depending on the reaction temperature (Supplementary Fig. 8). Thus, the higher chemical selectivity obtained during light-induced rWGS implies a unique pathway for chemical bond activation that is based on effective hot-carrier injection from photocatalyst to adsorbate(s) rather than photothermal heating. A recent demonstration of plasmonic photothermal heating for rWGS on Au/ZnO nanostructures also reported the simultaneous formation of both CH_4_ and CO under very high laser intensities (onset laser intensity of 25 W cm^–2^), consistent with our thermally driven rWGS results^23^. Plasmon-induced hot carriers have the potential to selectively manipulate the individual elementary steps of chemical reactions. By tuning the energy of hot carriers to match specific electronic states and/or vibrational modes of adsorbates, selectivity of chemical conversion can be improved and new mechanistic pathways can be opened^27^.

Plasmon-induced carrier-driven rWGS on Al@Cu_2_O is not only supported by higher product selectivity, but also by higher yields under illumination at comparable temperatures. The average steady-state surface temperature of 175 °C (Fig. 3b) under illumination at our maximum visible light intensity of 10 W cm^–2^ is below the 200 °C onset temperature of product formation in thermal-driven rWGS (Fig. 3d) but achieves a significantly higher overall reaction rate. This indicates that the strong interaction of plasmonic nanoparticles with incident photons at resonance frequency opens up new pathways for more efficient and selective chemical transformations under low operating temperatures.

**Fig. 4:**
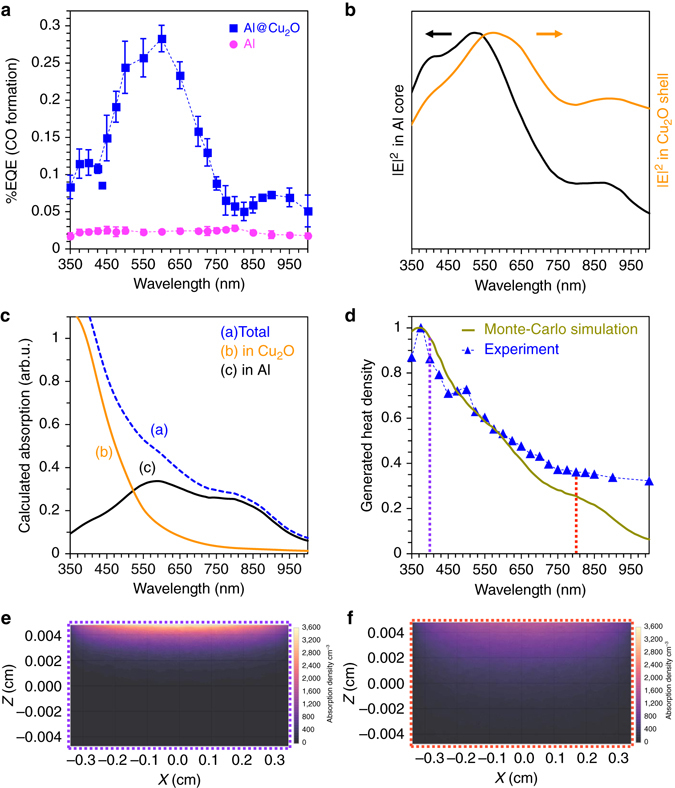
Distinguishing between plasmon-induced carrier-assisted and photothermal heating for rWGS on Al@Cu_2_O. **a** The measured EQE as a function of illumination wavelength for oxide-supported Al@Cu_2_O compared to Al vs. illumination wavelength. **b** Numerically calculated local electric field strength |*E*(*r*)|^2^ in Al core (left axis) and Cu_2_O shell (right axis). **c** A simulated absorbed fraction in 100 nm diameter Al core, 15 nm thick Cu_2_O shell and total structure placed on γ-Al_2_O_3_ in air. **d** Comparison between experimentally measured heat density and ensemble Monte-Carlo simulation vs. illumination wavelength for oxide-supported Al@Cu_2_O nanoparticles in air. **e**, **f** The absorption efficiency of oxide-supported Al@Cu_2_O nanoparticles at **e** 400 nm and **f** 800 nm obtained from ensemble Monte-Carlo calculation. The scales in **e** and **f** are based on the sample holder dimensions and volume of oxide-supported Al@Cu_2_O nanoparticles loaded into the sample holder. The calculated heat density corresponding to 400 and 800 nm are assigned in **d**

To elucidate the origin of efficiency enhancement observed for Al@Cu_2_O antenna-reactor nanoparticles compared to pristine Al NCs, we performed wavelength-dependent rWGS between 350 and 1,000 nm. It was found that coating Al NCs with a Cu_2_O shell substantially enhanced the EQE (Fig. 4a; also see Fig. 2b). This catalytic enhancement is particularly pronounced around the dipolar plasmon resonance of Al@Cu_2_O at ~570 nm. This and the results shown in Fig. 3 support a plasmon-enhanced mechanism for driving rWGS.

To further corroborate plasmon-induced carrier-assisted rWGS under illumination, we compared the wavelength-dependent study of CO formation (Fig. 4a) with local electric field enhancement, calculated by FEM, and the trend of wavelength-dependent heat density generation, as measured experimentally and calculated with a Monte-Carlo simulation. Previous theoretical^28^ and experimental^29^ studies have shown that the rate of carrier generation from plasmon decay is directly proportional to the plasmon-induced internal electric field enhancement. Thus, in order to gain a more rigorous understanding of plasmon-induced carrier generation in Al@Cu_2_O, we integrated the local electric field enhancement (|*E*(*r*)|^2^) in the Al core and Cu_2_O shell (Fig. 4b). The local electric field enhancement inside the Al core determines hot-carrier formation from non-radiative plasmon decay. These electrons can transfer into the Cu_2_O shell, where they will be longer lived than in the metal due to the reduced electron–electron scattering in a semiconductor^30^. The plasmon induced external field enhancements can also directly induce optical transitions (inter-band and/or sub-band transitions) in the Cu_2_O layer.

Figure 4b shows local electric field enhancement spectrum integrated over the volume of mean-free path of the Al core^31^ (within *r* = 50 nm) and over the entire volume of the 15 nm thick Cu_2_O shell, which should remain within the reported values for the carrier diffusion length in Cu_2_O^32^. We found that calculated |*E*(*r*)^2^| in both the Al core and in the Cu_2_O shell (Fig. 4b) qualitatively reproduce the measured EQE spectrum (Fig. 4a). The weak peak at 400 nm that appears in both Figs. 4a and b is due to excitation of the Al quadrupolar plasmon mode. The weak feature at 900 nm is due to the dispersion of the real part of the Al permittivity (interband transition). We note that the magnitude of the near-field induced carrier generation in the Cu_2_O shell depends on the density of states on the exposed surfaces^33^. FEM simulations do not account for imperfections, but the highly polycrystalline nature of the synthesized Cu_2_O shell (Fig. 1c) introduces defects within the crystal lattice. It is likely that near-field energy transfer can generate carries at energies below the band gap of Cu_2_O, i.e., the sub-band gap transitions at 900 nm. The dual mechanisms of carrier generation either in the Al core with subsequent injection into the Cu_2_O shell and/or the direct excitations of hot carriers in the Cu_2_O shell, as suggested by the calculations in Fig. 4b both provide enhanced hot carrier densities for chemical transformations. To separate the influence of the plasmonic core, a similar structure was modeled, where the Al core was replaced with a dielectric Al_2_O_3_ sphere of the same size. In this case, the calculated local electric field enhancement in the volume of the Cu_2_O shell did not reproduce the features in the experimental EQE spectrum (Supplementary Fig. 9). This validates the role of the Al antenna for carrier generation within Al@Cu_2_O through plasmon decay.

We also simulated the total photon absorption within Al@Cu_2_O, as well as the absorbed photon fractions in each component (Fig. 4c), and found that the simulated total absorption spectrum does not match the experimentally measured EQE spectrum for Al@Cu_2_O (blue line, Fig. 4a). The increased total absorption at wavelengths below 550 nm in Fig. 4c is attributed to interband absorption in Cu_2_O. The EQE in this wavelength region, however, follows the simulated absorbed fraction within the Al core rather than the Cu_2_O shell or the total structure (Fig. 4c), implying that carrier generation at or near the band gap of Cu_2_O relies on the plasmonic Al core. The simulated absorbed photon fraction at wavelengths above 700 nm does not perfectly resemble the EQE spectrum as the peak at 900 nm, which is redshifted with respect to the interband transition of Al centered at 800 nm. In contrast, this peak has been predicted by our local electric field enhancement calculation in Fig. 4b.

The trend of wavelength-dependent heat density under illumination, as measured experimentally and calculated with a Monte-Carlo simulation, does not reproduce the measured EQE spectrum of Al@Cu_2_O (Fig. 4a). Therefore, the temperature increase due to photothermal heating is not responsible for chemical bond activation during photocatalytic rWGS. Rather, selective CO formation is driven by photogenerated carriers, as confirmed by calculated local field enhancement in Fig. 4b. The experimental wavelength-dependent heat generation within the photocatalyst for a constant number of photons at each wavelength shows excellent agreement with ensemble calculations obtained from Monte-Carlo simulations, demonstrating the validity of our theoretical model. The theoretical heat density is calculated using Monte Carlo light transport simulations, and is defined as the fraction of incident photons absorbed divided by the penetration depth of absorbed photons (see Methods and Supplementary Note 2)^26^. The reasons that the temperature increase observed in the experiment do not track the single-particle plasmon in the simulated absorption spectrum (Fig. 4c) are two-fold: the absorption efficiency of the ensemble is significantly different because of the presence of multiple photon scattering events, and the localization of absorbed power causes larger heat densities at shorter wavelengths (Figs. 4e and f).

## Discussion

**Fig. 5:**
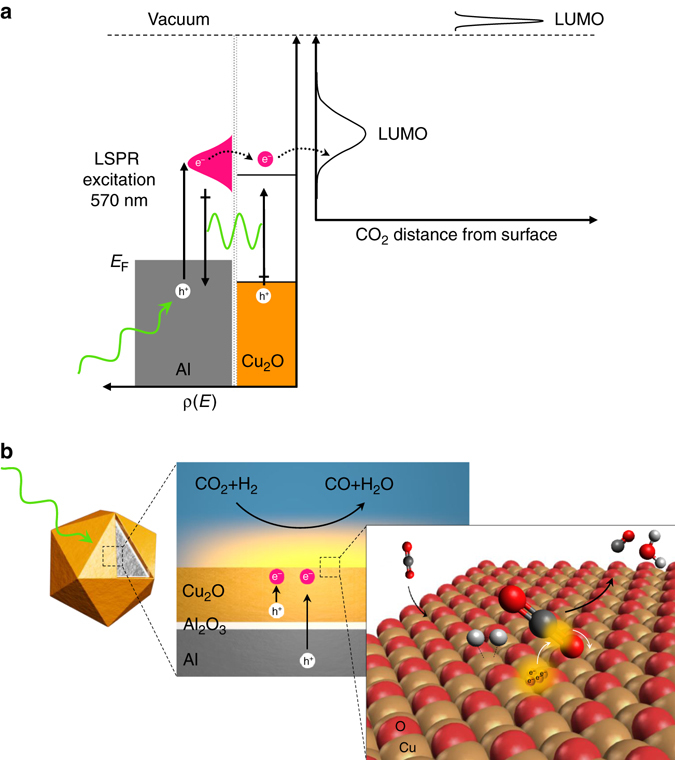
Structure and mechanism of plasmon-induced carrier-assisted rWGS on Al@Cu_2_O. **a** Energy band diagram of Al@Cu_2_O for plasmon-mediated carrier generation for injection into unoccupied state of CO_2_ for C–O bond activation. **b** Schematic of plasmon-induced carrier-driven rWGS on Al@Cu_2_O

Our results in Figs. 3 and 4 establish a plasmon-induced carrier-assisted rWGS. The higher efficiency of light-assisted rWGS on Al@Cu_2_O antenna-reactor heterostructures compared to pristine Al NCs can be attributed to concurrent enhancement in surface catalytic activity and the rate of carrier generation. An energy diagram and a schematic of the proposed elementary steps for plasmon-induced carrier-assisted rWGS on Al@Cu_2_O are illustrated in Fig. 5. Experimental and theoretical studies^34, 35^ have reported an adiabatic electron affinity of approximately –0.6 eV±0.2 per CO_2_ molecule in gas-phase. The negative electron affinity explains the difficulty of electron injection into CO_2_ to form $\rm CO_2^ -$, the first and the most difficult step in CO_2_ activation^36^. However, adsorption of CO_2_ on Cu_2_O surfaces results in charge redistribution with charge transfer from the surface to adsorbed CO_2_ that induces CO_2_ polarization, and consequently the energy barrier for transient electron transfer to unoccupied states of adsorbed CO_2_ should reduce^33, 36, 37^.

While several modes and geometries for adsorption of CO_2_ on Cu_2_O surfaces exists, previous density functional theory calculations with Hubbard corrections (DFT+U) suggested that unsaturated surface copper atom (Cu_CUS_) in Cu_2_O(111):Cu, which is the least thermodynamically stable Cu_2_O surface, promotes strongest CO_2_ adsorption^33, 37, 38^. The initial CO_2_ adsorption occurs in a linear-tilted configuration in which the oxygen atom (Lewis base) in CO_2_ binds to Cu_CUS_ (Lewis acid) active site. Calculations have also shown that hydrogen can adsorb dissociatively on Cu_CUS_ forming δ^+^H and δ^–^H adatoms^38^. On the contrary, spontaneous dissociation of CO_2_ on Cu_2_O under ambient conditions is not energetically favorable due to high activation energy barrier to the transition state^38^. Accordingly, no product formation was measured without external illumination at room temperature in our experiments (Supplementary Fig. 5).

Adsorbed CO_2_ can be activated through a plasmon-induced carrier-assisted mechanism. Photogenerated carriers from LSPR excitation in general overcome the activation energy barrier and transiently populate antibonding orbitals of adsorbed CO_2_ creating the transient negative ion (TNI)$\rm CO_2^ -$, which could subsequently relax into an excited vibrational state in the ground state by releasing the electron back to Cu_2_O[5]. The net result is that the photogenerated hot-carriers can deposit their energy into the vibrational mode of C–O causing vibrational excitation and C–O bond elongation. Since the unoccupied molecular orbitals of CO_2_ are hybridized orbitals between the C and two O orbitals, the hot-carrier transfer into these orbitals can directly facilitates C–O bond dissociation, initiating the rWGS. The remaining oxygen on the surface Cu_CUS_ atom interacts with two previously dissociated hydrogen adatoms on the surface to form water. The desorption of water should be enhanced by energetic carriers, which otherwise should adsorb dissociatively on Cu_2_O(111)^38^.

The unique selectivity observed during light-induced processes on Al@Cu_2_O can be explained based on plasmon-induced selective C–O bond activation and desorption of CO* from reactive surface sites. The net energy transfer from hot-carriers to intermediates on the surface can induce a nonthermal desorption of molecules^39^. Plasmon-induced selective CO formation was found to be independent of surface chemistry as it was observed for light-induced rWGS on both pristine Al and Al@Cu_2_O photocatalysts. Similarly, selective CO formation was previously reported during plasmon-enhanced CO_2_ conversion on Au and Ag nanostructures^25, 40^. In addition, varying the ratio of H_2_ to CO_2_ in the gas steam had negligible effect on product selectivity for light-induced rWGS, which continuously exhibited selectivities towards CO of over 99.3% (Supplementary Fig. 10). Furthermore, selective CO formation was observed during light-induced rWGS in a close-batch reactor (Supplementary Fig. 11, and Supplementary Method 3), suggesting that selective desorption of CO* from reactive surface sites is independent of space velocity of gas steam. Based on these observations we surmise that direct C–O bond dissociation plays a major role in the light-induced rWGS reaction, and that the principal role of H_2_ is to seize the oxygen adatoms to form water and maintain an active catalytic surface, which otherwise should be deactivated in the absence of H_2_ (Supplementary Fig. 12). Despite the 2–4 nm self-limiting amorphous Al_2_O_3_ layer surrounding the Al core, tunneling of the hot carrier to the oxide surface is still viable due to a high density of defect states in the amorphous Al_2_O_3_ layer. The direct dissociation of adsorbed CO_2_ on pristine Al NCs under visible light illumination at ambient conditions provides evidence for this hot-carrier tunneling mechanism (Supplementary Fig. 12). The formation of CO in absence of H_2_ justifies the transfer of hot-carriers into unoccupied orbitals in CO_2_ for C–O bond dissociation. Photodissociation of non-adsorbed CO_2_ would not be possible, which requires incident photons at vacuum ultraviolet energies (>10 eV)^41^.

From a carrier generation perspective, the higher efficiency of light-assisted rWGS on Al@Cu_2_O antenna-reactor heterostructures compared to uncoated Al nanocrystals can be attributed to enhancement in the rate of carrier generation in Al@Cu_2_O. In the absence of the Cu_2_O layer (uncoated Al) only the non-radiative damping process can contribute to carrier generation. This carrier generation pathway, however, is not efficient for 100 nm Al NCs used in this study as the main damping pathway for nanocrystals of this size is through a radiative process. The FEM calculations in Fig. 4b, clearly shows that the enhanced carrier generation in Al@Cu_2_O can be attributed to non-radiative plasmon decay in the Al core and near-field excitation of carriers in the Cu_2_O. Quantitatively ascertaining the ratio of carriers generated by each pathway is non-trivial. The efficiency of resonance energy transfer primarily depends on the overlap between the LSPR absorption and the optical transitions in nearby semiconductors^10^. Calculated absorbed photon fractions (Fig. 4c) show significant LSPR overlap with the band edge absorption of Cu_2_O, which can induce interband/sub-band transitions in the semiconductor shell. The localized resonance energy transfer within a 15 nm thick Cu_2_O shell allows for carrier generation at volumes much lower than the bulk carrier diffusion lengths^32^, thereby significantly decreasing the carrier recombination rate. This is particularly important because, based on absorption coefficient values^42^, carrier generation from interband transitions in Cu_2_O is being achieved at absorption depths much higher than its carrier diffusion length. This is also evidenced by our results where the obtained EQE at the band gap of 15 nm thick Cu_2_O (below 500 nm) is much lower than that of at LSPR frequency near the band edge of Cu_2_O (see Figs. 4a and c). In addition to plasmon-induced resonance energy transfer, the proper energy band alignment of Al and Cu_2_O (Fig. 5a) satisfies the requirement for plasmon-induced hot electron transfer to the Cu_2_O layer. Therefore, Cu_2_O should further improve the efficiency of hot-carrier injection to adsorbates by increasing the lifetime of excited-states by facilitating their extraction from the Al core at the interface of native oxide layer. Compared to Au or Ag, Al has a higher Fermi energy and a favorable band structure so that Al has non-competing direct excitation pathways (interband transitions) at higher photon energies. This is important for effectively utilizing hot-carriers to drive chemical reactions of molecules whose antibonding orbitals are only accessible by highly energetic carriers.

Recent work in the area of plasmon-enhanced photocatalysis has demonstrated two resonance energy transfer mechanisms from plasmonic particle to an adjacent semiconductor: (i) near-field enhancement, (ii) dipole-dipole coupling. The conventional energy transfer mechanism lies in near-field electromagnetic enhancement associated with radiative plasmon decay for enhancing optical transitions in the nearby semiconductor^4^. An alternative scenario has been recently proposed for non-radiative “plasmon induced resonance energy transfer” (PIRET) based on coupling of large plasmon dipole to the dipole of electron-hole pair in semiconductor for direct carrier excitation^43^. In principle, both mechanisms operate under similar conditions that rely on the plasmonic dipole and local electric field enhancement |*E*(*r*)|^2^ to induce carrier excitation in the adjacent semiconductor. Here, we interpret our FEM simulation results in the context of near-field enhancement mechanism, although the existence of a PIRET mechanism is also plausible. However, due to the difference between electronic band structure of Al and Au (previously studied for PIRET in Au@Cu_2_O), a more detailed study will be required in the future in order to provide a better insight into the PIRET mechanism in enhanced photocatalytic activity observed for Al@Cu_2_O system.

We have presented a paradigm in plasmon-mediated heterogeneous catalysts for light-driven, selective rWGS—a plasmonic heterostructure that exclusively utilizes non-precious, earth-abundant elements. The rWGS reaction, while central to the chemical industry, also mitigates one of humanities greatest threats: anthropogenic CO_2_. Our study presents a low cost solution for turning CO_2_, an unwanted byproduct, into a valuable reagent for the production of other high-value chemical products. Our measured external quantum efficiency on Al@Cu_2_O of 0.3% is, to the best of our knowledge, the highest reported value for plasmon-induced CO_2_ conversion. We demonstrate that rationally and predictively designing the Al@Cu_2_O antenna-reactor nanoparticles provides independent control of both the catalytically active surfaces and plasmonic light absorption. Separating catalytic (Cu_2_O) and light harvesting (Al) functions into different materials is the key to achieving higher quantum efficiencies in chemical conversion. We have shown that the Al@Cu_2_O antenna-reactor complexes also yield increased selectivity over traditional thermal-driven reactions. Utilizing hot carriers generated from plasmon decay can greatly influence reactivity and selectivity under mild conditions. The present work represents a significant step toward understanding how plasmonic photocatalysts can be used as sustainable alternatives to traditional energy-intensive heterogeneous catalytic processes.

## Methods

### Synthesis of photocatalyst

Al NCs with an average diameter of 100 nm with a 2–4 nm self-limiting oxide surface layer were chemically synthesized according to our previously published protocol^19^ with minor modifications. Briefly, 5 ml of anhydrous tetrahydrofuran (THF) and 15 ml of anhydrous 1,4-dioxane (Sigma-Aldrich) were mixed in a 100 ml dry Schlenk flask under an Ar atmosphere at 40 °C. Under stirring, 6.5 ml of N,N-dimethylethylamine alane (0.5 M in toluene, Sigma-Aldrich) was injected into the reaction vessel, followed by rapid injection of 0.5 ml of 2 wt% Ti(OiPr)_4_ in toluene. The color of the solution turned to brown within a few seconds, and to gray within an hour, indicating formation of Al NCs. The reaction was allowed to proceed for 2 h, before being removed from the heat source. 1 ml of oleic acid was injected into the mixture to quench the reaction. The as-synthesized nanoparticles were isolated by sonication and centrifugation (2,000 g) in dry toluene, followed by three cycles of washing and centrifuging in 2-propanol (IPA). Finally, Al NCs were dispersed in IPA and the solution purged by Ar and stored at room temperature for future use.

For the synthesis of Al@Cu_2_O, 2.5 ml of as synthesized Al NCs (1 mg ml^–1^ in IPA) were transferred to an oven-dried Schlenk flask and the total volume of the solution adjusted to 10 ml using IPA. The reaction solution was degassed at room temperature for about an hour and then under Ar atmosphere the flask was heated to reflux. While refluxing, 1 ml of 0.01 M fresh Cu (II) acetate (99.999% trace metal-basis, Sigma-Aldrich) in dry acetonitrile was rapidly injected into the reaction with constant stirring. The reflux continued for 2 h to yield Al@Cu_2_O nanoparticles. The as-synthesized nanoparticles were isolated by centrifuge at 2000 g and washed three times with IPA, and finally dispersed in IPA. For control experiment, we have prepared Cu_2_O through an alcoholic reduction method similar to the technique used to grow a Cu_2_O layer around Al.

### Preparation of catalyst and reactor set-up

The photocatalysts were prepared by mixing a homogenous dispersion of plasmonic particles with a commercial γ-Al_2_O_3_ support (Alfa Aesar) at 5 wt% loading with respect to the oxide support. Briefly, the proper amount of oxide support was added to the nanoparticle solution in IPA. The mixture was shaken thoroughly to obtain a homogenous mixture and then centrifuged to remove the excess IPA. The remaining solid pellet was dried overnight under vacuum. The powdered catalyst/Al_2_O_3_ mixture was ground using a mortar and pestle to homogenize the dry catalyst before being loaded into the reaction chamber. The percentage of Cu in the Al@Cu_2_O supported oxide catalyst was as low as 1 wt% based on quantifying the Cu precursor. For measuring the photocatalytic activity, about 20 mg of oxide-supported plasmonic nanoparticles were loaded into a customized stainless steel chamber flow fixed-bed reactor (Harrick Scientific Product Inc.), equipped with a precise thermocouple and a quartz window for illumination. At a total pressure of 1 atm, high purity H_2_ (99.999% Matheson trigas) and CO_2_ (99.999% Matheson trigas) were continuously flowed into the reaction chamber at a total flow rate of 10 s.c.c.m.. For online monitoring of product formation, the reaction chamber was connected to a gas chromatograph (Shimadzu) equipped with a pulsed discharge helium ionization detector (PDHID) (connected to a molecular sieve 13× column) and a flame ionization detector (FID) (connected to a fused silica PLOT column).

### Photocatalytic and thermal processes

Photocatalytic and thermaocatalytic measurements were performed at a 1:1 ratio of CO_2_:H_2_ (total flow rate of 10 s.c.c.m.). The CO_2_:H_2_ stoichiometry of 1:1 was sustained throughout all experiments except when specified to the contrary (Supplementary Fig. 10). Thermal activity measurements were conducted in the absence of light, and by heating the chamber with a precise temperature controller (Harrick Scientific Product Inc.). For photocatalytic experiment without external heating, a supercontinuum fiber laser (Fianium, 400–850 nm, 4 ps, 80 MHz, see Supplementary Fig. 5 for laser spectrum) was used as the visible excitation source. For Visible light studies, a lens (Thorlab, 150 mm focal length) was used to focus the light to a 1.5-mm spot size onto the photocatalyst, through the quartz reactor window. Wavelength dependence measurements were obtained over the range of 350–1,000 nm. Monochromatic light from a tunable Ti:sapphire laser (Coherent, Chameleon Ultra II, 150 fs, 80 MHz, bandwidth ~10 nm) was utilized the wavelength range of 680−1,080 nm, and coupled to a second harmonic generator (Angewandte Physik und Elektronik GmbH) for the output wavelength range of 350–530 nm. For wavelength dependence measurements in the range of 500–700 nm, we used the supercontinuum fiber laser and the appropriate bandpass filters (Edmund, bandwidth ~50 nm, 80 MHz). The average power of the incident beam was fixed at 130±2 mW for each wavelength. A 90° off-axis parabolic mirror (PFL 76.2 mm) was used to focus the light to a sub-millimeter spot to avoid chromatic aberration.

The EQE was calculated from the rate of CO formation at incident photon energy impinging the surface (EQE_CO_ = 2 × rate [CO] (μmol cm^–2^ s^–1^)/photon flux (μmol cm^–2^ s^–1^)). For visible light illumination, an average photon energy at 700 nm was used according to the light source spectrum (Supplementary Fig. 5). For Wavelength-dependent study the photon energy at each illumination wavelength was used for EQE calculations.

### Photothermal heating measurements

A highly sensitive thermal imaging camera (FLIR A65) was used for high-resolution (640 × 512 pixels) photothermal imaging and as a temporal monitor of temperature variation. Photothermal measurements were performed under white light illumination and as a function of illumination wavelength. The sample was exposed to the lab atmosphere environment during illumination (due to limited transparency of the quartz reactor window at thermal infrared wavelengths). The thermal camera was positioned above the sample at a slightly tilted angle with a few centimeters off from the center of the stage to allow for top illumination of the sample.

### Monte-Carlo simulations

Monte Carlo simulations were performed to model light transport using a customized code that was verified using both analytic and computational standards. Each calculation included the simulation of between ~1 and 4 × 106 photon trajectories, depending on the simulation wavelength. Each calculation was terminated when 106 absorbed photons was reached for each incident wavelength, keeping the statistical error for the calculations relatively constant. The simulation domain approximated the experimental photocatalytic geometry using a rectangular cell of dimensions 8 × 8 × 0.1 mm. A nanoparticle concentration of 1.14 × 1014 cm^–3^ (5 wt% loading of particle) was multiplied by the appropriate optical cross sections for the photocatalyst nanoparticle obtained from FEM calculations, to obtain scattering and absorption coefficients as input. The scattering coefficient ranged from 55 to 673 cm^–1^, while the absorption coefficient ranged from 31 to 520 cm^–1^. The alumina support was assumed to be a purely scattering medium with a scattering coefficient of 70 cm^–1^. Each photon was simulated until it was either absorbed or exited the simulation domain. Photon scattering followed a dipole scattering cos^2^(θ) distribution. Upon absorption, the position of the photon was saved in order to calculate the photon absorption distributions. Absorption efficiencies were calculated by dividing the number of photons absorbed by the total number of photons simulated. When needed, the obtained spectra were normalized to take into account the different photon energies for different wavelengths of light.

### Electromagnetic simulations

Modeling of the Al@Cu_2_O antenna-reactor nanoparticles was performed using the Finite Element Method (FEM, COMSOL Multiphysics 4.2a). The dimensions of nanoparticles were chosen to be identical to the nominal sizes of synthesized structures apparent from the TEM images. The particle was embedded in a γ-Al_2_O_3_ medium, which was modeled as a 1:1 mixture of air and Al_2_O_3_, and perfect matched layers (PMLs) were adopted to simulate the infinite environment. A 3 nm Al_2_O_3_ separation layer was included in the simulations to account for the surface oxide of Al. The dielectric responses of all materials were taken from tabulated data^44^. The optical absorption was calculated by integrating the Ohmic loss within the nanostructures.

### Data availability

The data that support this study are available from the corresponding author on request.

## Electronic supplementary material

Supplementary Information

TPR
