# Supplementary material for: Plasmon-induced selective carbon dioxide conversion on earth-abundant aluminum-cuprous oxide antenna-reactor nanoparticles
Source: Nat Commun. 2017 Jun 21;8:27. doi: 10.1038/s41467-017-00055-z (PMC5479834; doi:10.1038/s41467-017-00055-z)
Supplement: Supplementary file 1 — Supplementary Information [file 41467_2017_55_MOESM1_ESM.pdf]

File Name: Supplementary Information
Description: Supplementary Figures, Supplementary Notes, Supplementary Methods and Supplementary References

File Name: Peer Review File
Description:

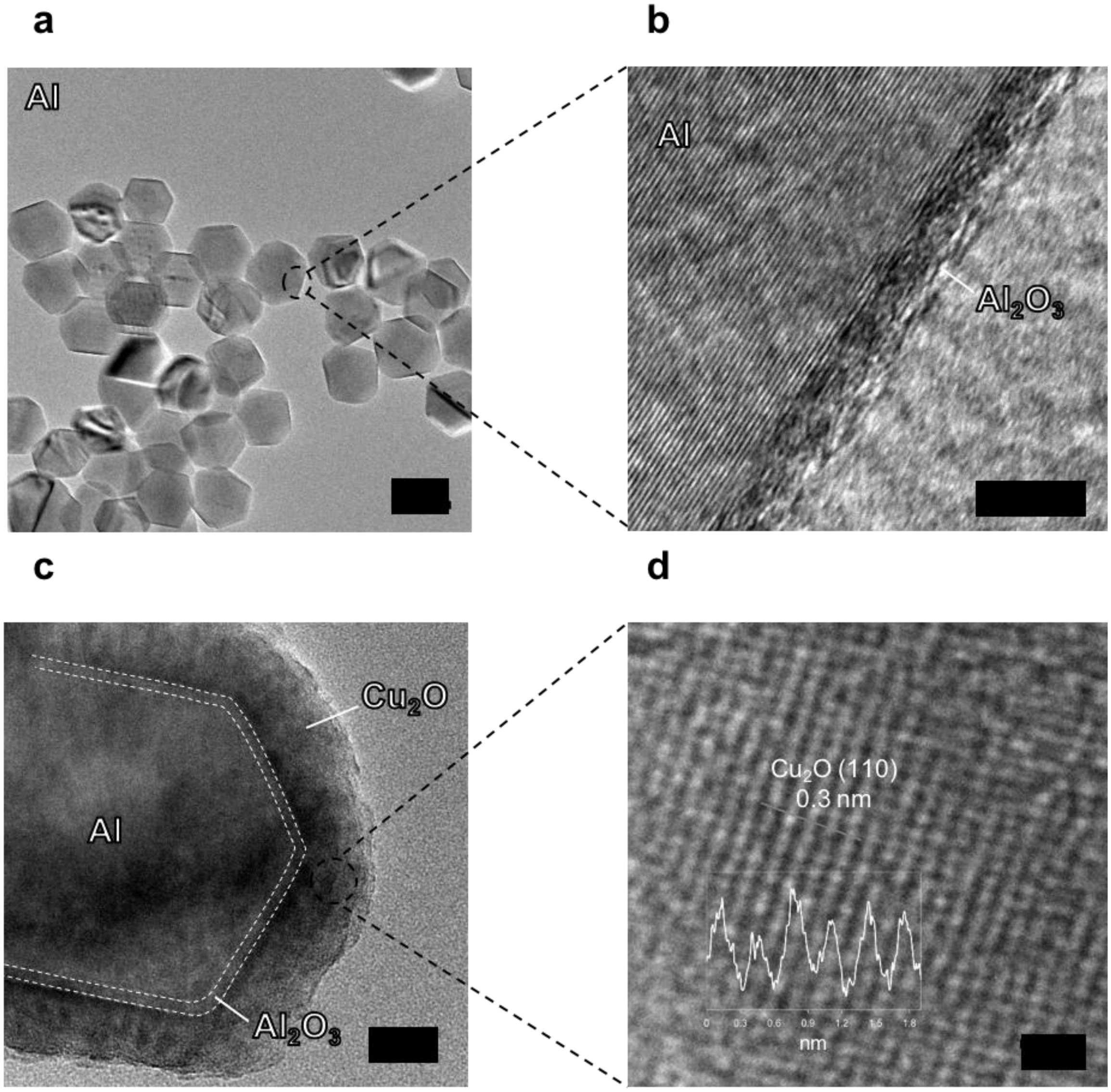

Supplementary Figure 1. Electron Microscopy characterization. High resolution TEM images of Al and of Al@Cu_2O nanoparticles. Each Al nanocrystal is surrounded by a 2-4 nm amorphous, native Al_2O_3 layer that also separates the Al core from the Cu_2O layer in the Al-Cu_2O nanoparticles. The Cu_2O shell was grown uniformly around Al core. HRTEM image of the Cu_2O layer shows lattice fringe with d-spacing of about ~0.3 nm corresponding to Cu_2O (110). Scales bars in (a) is 100 nm, in (b) is 5nm, in (c) is 20 nm, and in (d) is 1 nm.

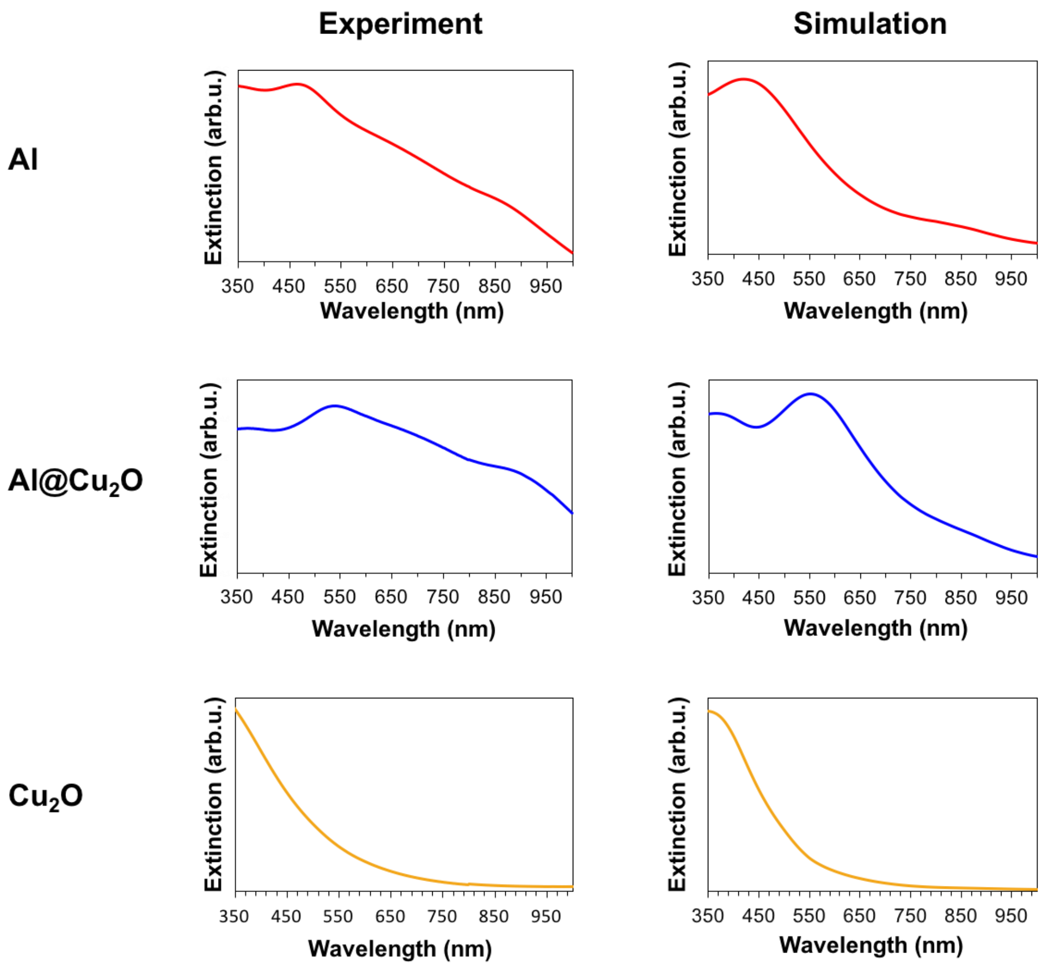

Supplementary Figure 2. Optical characterization of Al, Al@Cu_2O and Cu_2O. Experimental (left) and theoretical (right) UV-Vis extinction spectra of Al NCs, Al@Cu_2O and Cu_2O in IPA. Pristine Al NCs show a dipolar LSPR around 460 nm that redshifts to around 550 nm after growth of the Cu_2O shell (typical thickness of the is about 15-20 nm) due to real part of the Cu_2O permittivity. Extinction spectra in the wavelength range from 350 nm to 1000 nm of the nanoparticles were recorded by Cary 5000 UV-Vis-NIR Spectrometer.

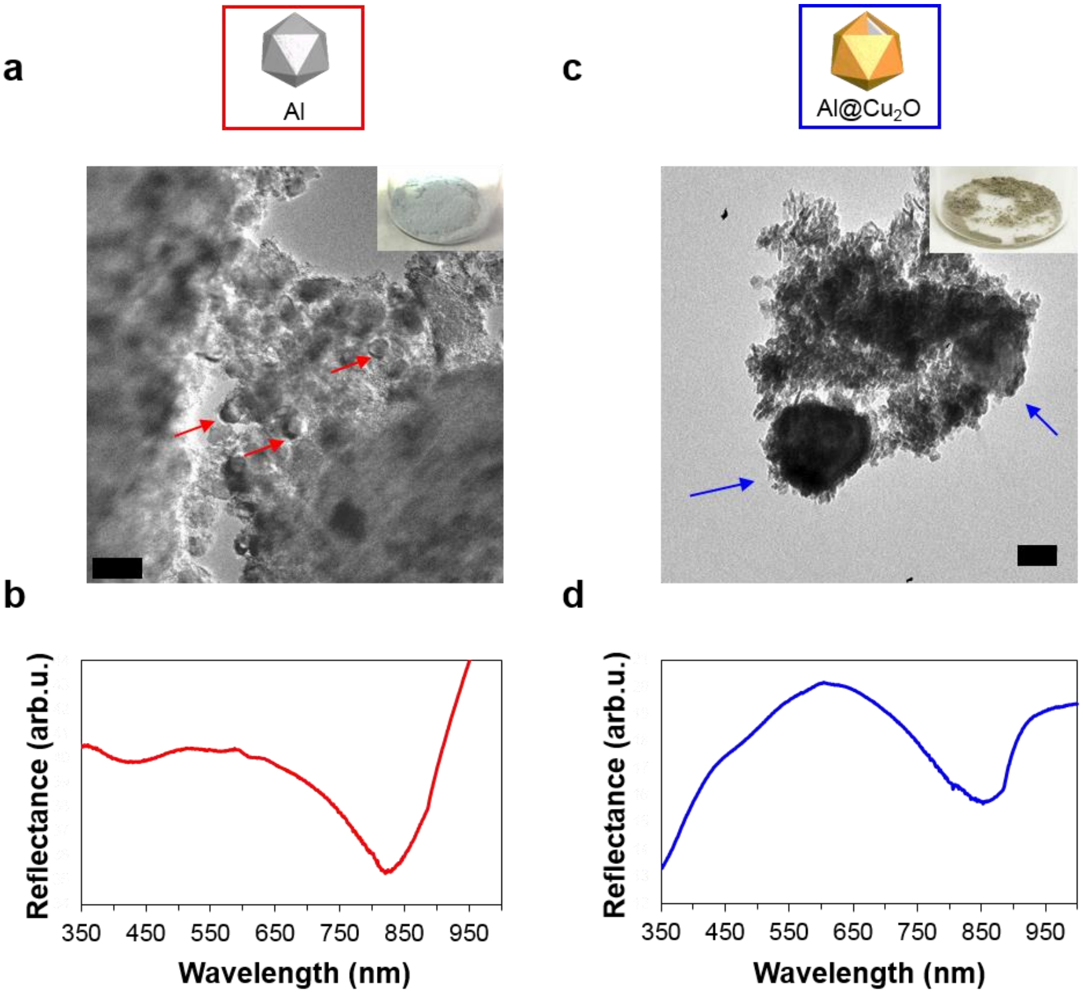

Supplementary Figure 3. TEM and optical characterization of oxide-supported Al and Al@Cu_2O. TEM images and corresponding UV-Vis diffuse reflectance spectra of **(a,b)** Al NCs/γ-Al_2O_3 and **(c,d)** Al@Cu_2O/γ-Al_2O_3 mixtures with dipolar plasmon resonances at about 475 nm and 575 nm, respectively. The Al interband transition appears as a dip in the reflectance spectra. The scale bar in (a) and (c) are 200 nm and 50 nm, respectively.

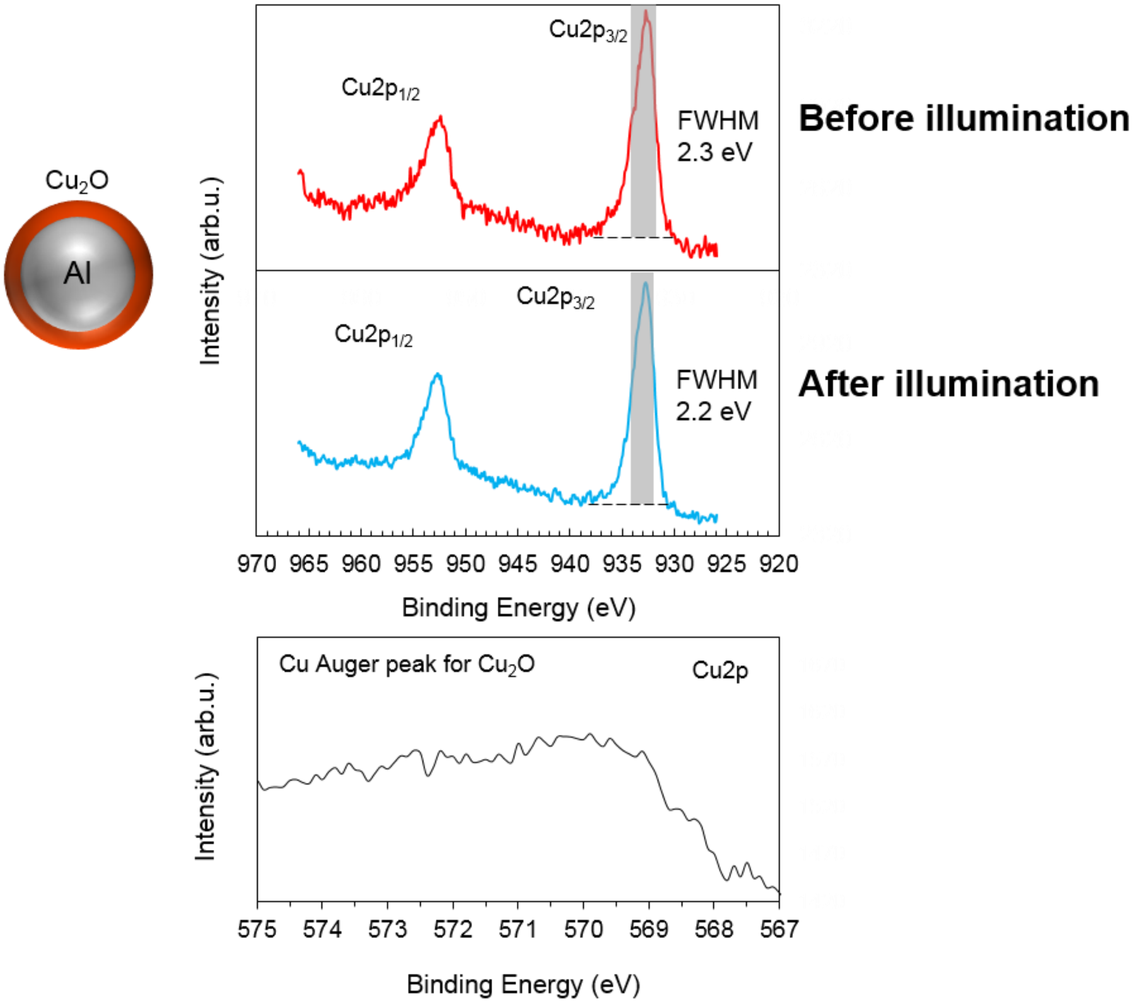

Supplementary Figure 4. X-ray photoelectron spectroscopy (XPS) characterization of Al@Cu_2O sample before and after illumination under reaction condition.

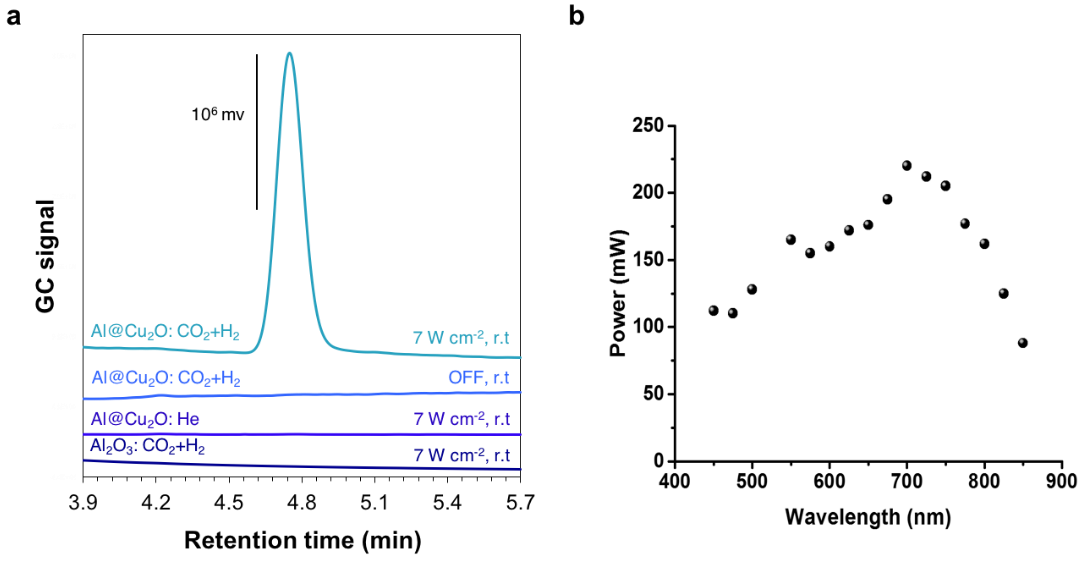

Supplementary Figure 5. Preliminary photocatalytic CO_2 hydrogenation and control experiments. (a) Typical gas chromatogram of the reaction chamber under various conditions. **(b)** Spectrum of the light source used for illumination in (a). CO formation was only detected during irradiation of $Al@Cu_2O$ nanoparticle/γ-Al_2O_3 mixture in CO_2 and H_2 (1:1, total flow rate of 10 sccm).

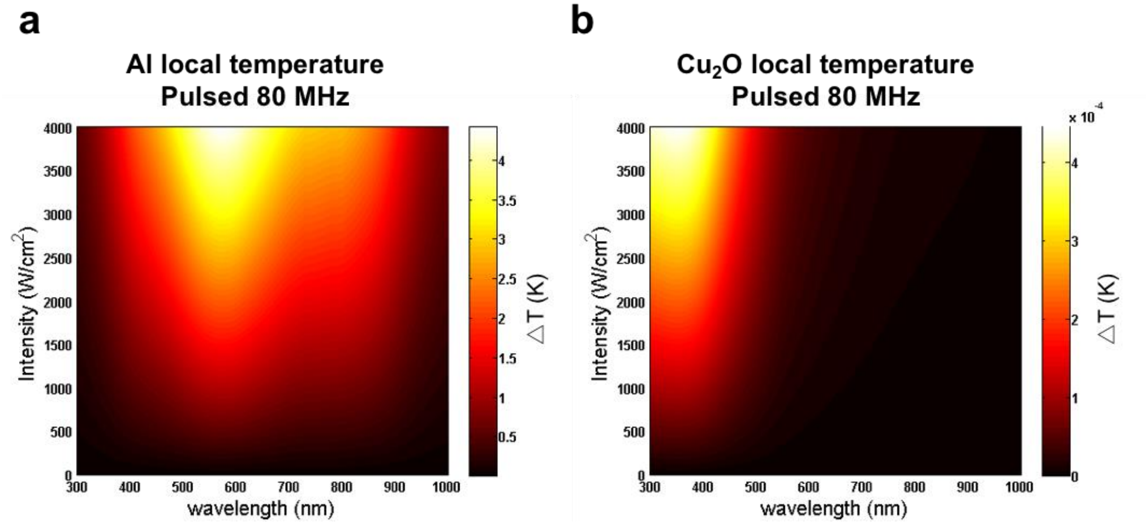

Supplementary Figure 6. Calculated local heating on single particle surface. (a) Calculated local heating on Al under illumination as a function of wavelength and laser intensity **(b)** Calculated local heating on Cu_2O under illumination as a function of wavelength and laser intensity.

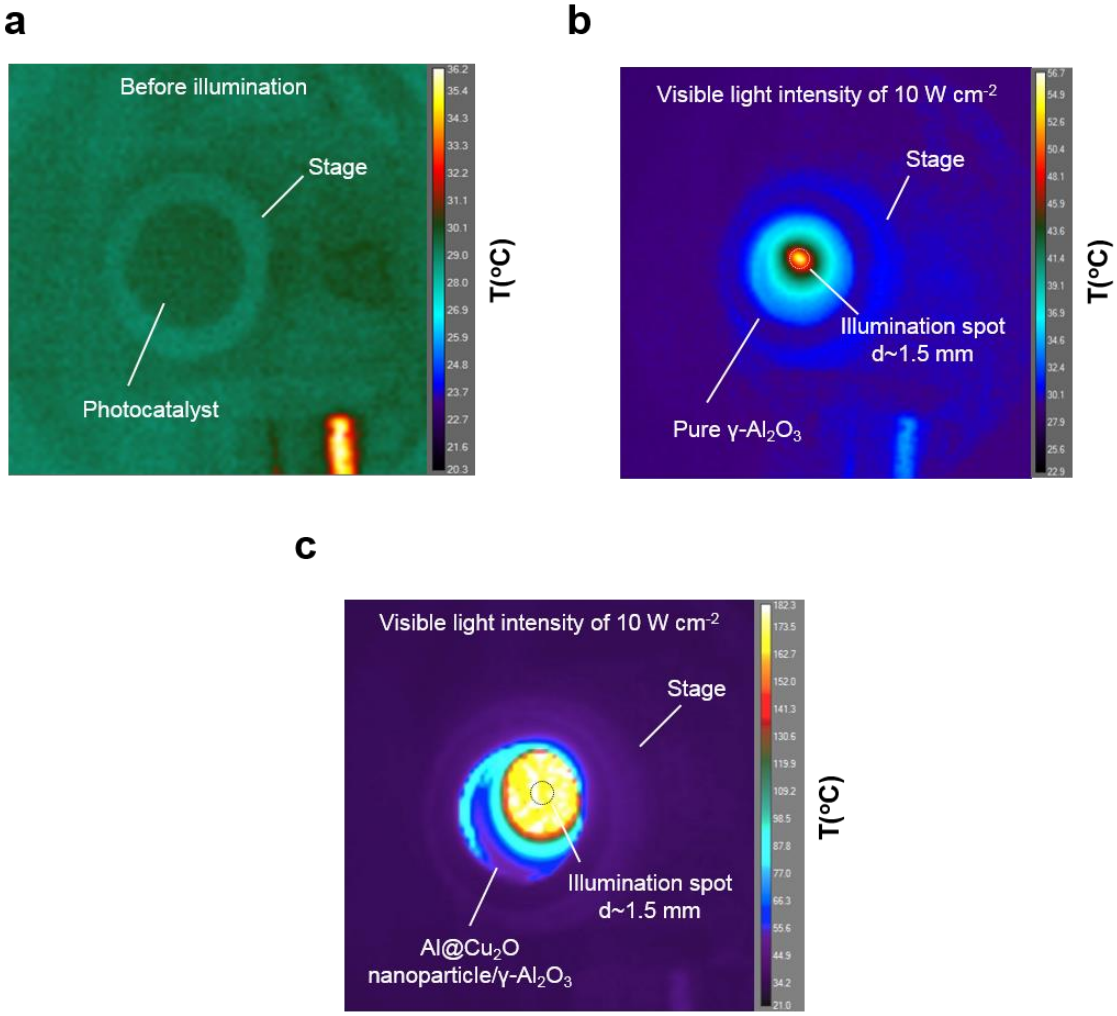

Supplementary Figure 7. Spatial high resolution photothermal imaging. (a) Before the illumination, the photocatalyst and stainless steel stage are in thermal equilibrium with room temperature. **(b)** Upon illumination with 10 Wcm^{-2} visible light, the temperature of pure oxide support reaches up to 55°C, while after loading plasmonic nanoparticles into the oxide support, the temperature rises up to 180°C under the same light intensity. **(c)** The area with high temperature increase in (c) is much large than that initial beam size due to multiple scattering events in medium by plasmonic particle surface. In (b), however, the heated area stays very close to the initial beam size in absence of plasmonic particle oxide support.

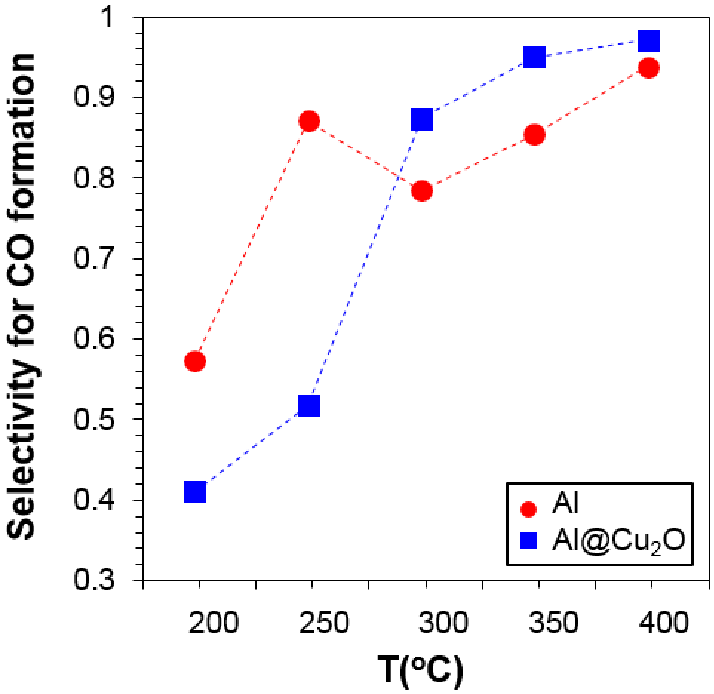

Supplementary Figure 8. Thermal-driven rWGS: CO/CH_4 selectivity as function of temperature.

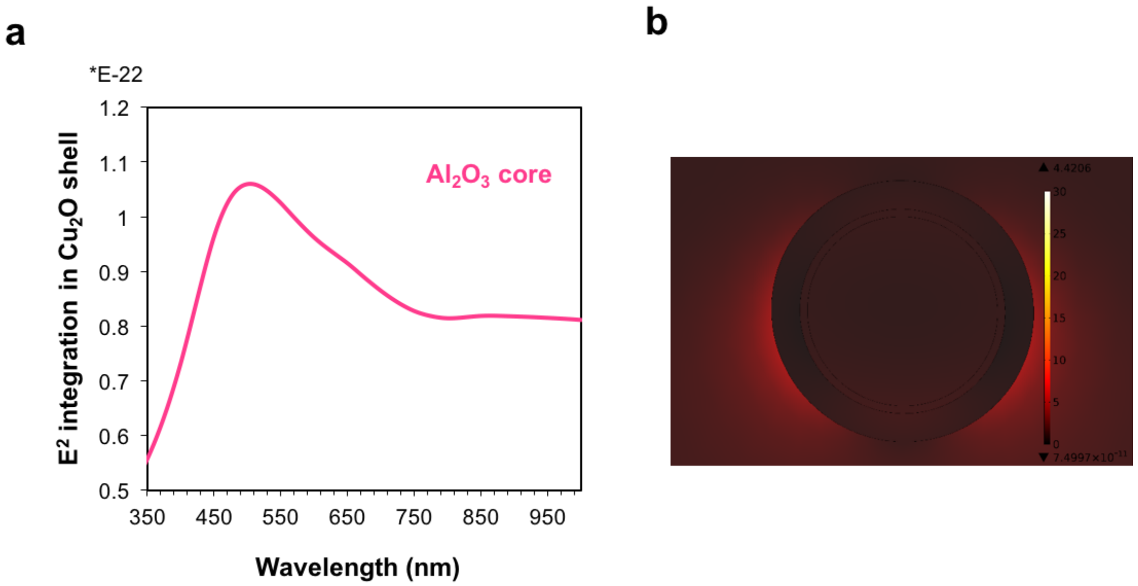

Supplementary Figure 9. Local field enhancement calculation for Al_2O_3@Cu_2O. (a) Local field enhancement $|E(r)|^2$ spectrum Integrated over the volume of 15 nm thick Cu_2O shell surrounding 100 nm Al_2O_3 sphere. As can be seen, unlike to the Al core of the same size (Fig. 3b), calculated $|E(r)|^2$ in Cu_2O shell surrounding Al_2O_3 core does not reproduce the features in measured EQE spectrum. **(b)** $|E/E_0|^2$ plot of Al_2O_3@ Cu_2O at 500 nm peak.

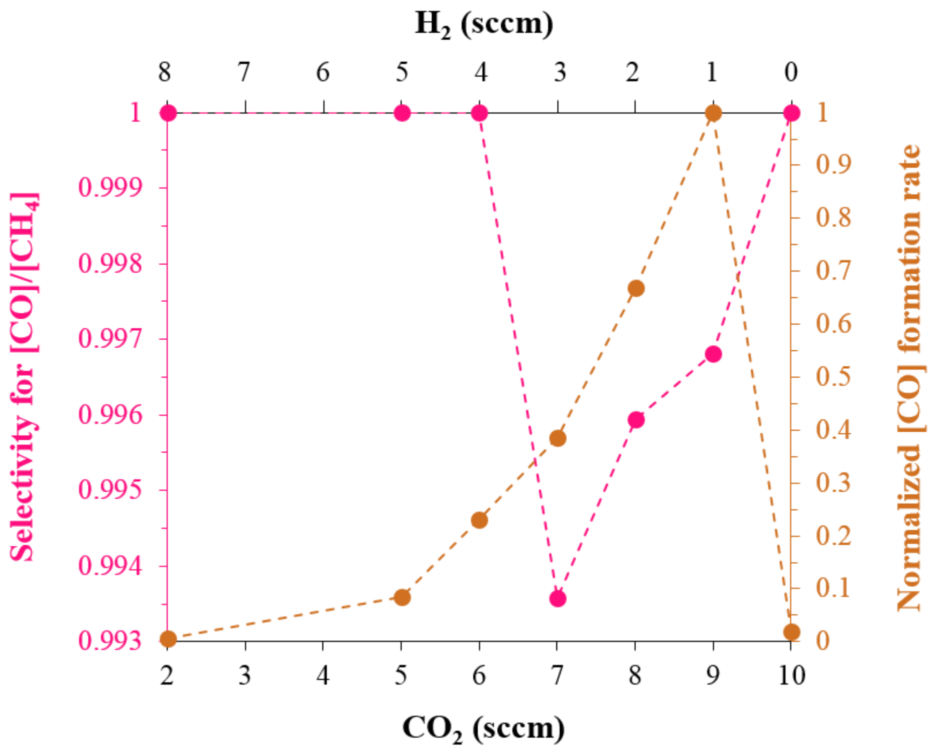

Supplementary Figure 10. Effect of variation in stoichiometric ratio of CO_2:H_2 on selectivity of product formation during plasmon-induced rWGS. Photocatalytic measurements were performed on Al@Cu_2O/γ-Al_2O_3 under visible light intensity of 8 Wcm^{-2}. Variation in the stoichiometric ratio of CO_2:H_2 was found to have negligible effect on the selectivity of CO formation during plasmon-induced rWGS. CO and CH_4 were found as the major and minor product, respectively, with CO/CH_4 selectivity of at least 99.35%. The total flow rate was adjusted to 10 sccm throughout the experiments to avoid pressure variation during GC analysis. The CO formation rate increased alongside increasing CO_2 content in the flow gas, but sharply decreased in absence of H_2 in the flow gas.

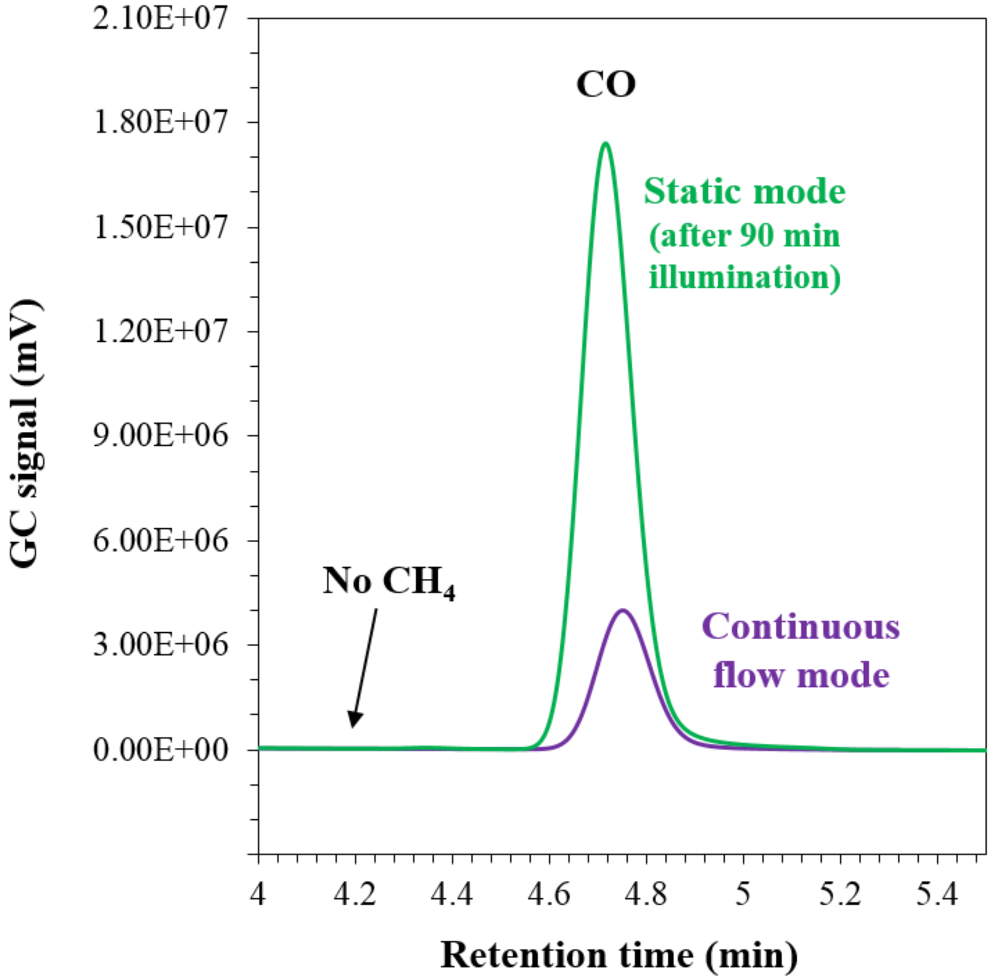

Supplementary Figure 11. Photocatalytic study of rWGS in static mode. Similar to flow packed-bed reactor conditions in which CO_2 and H_2 were continuously flowed into the chamber, CO was found as the product of CO_2 transformation in static mode configuration.

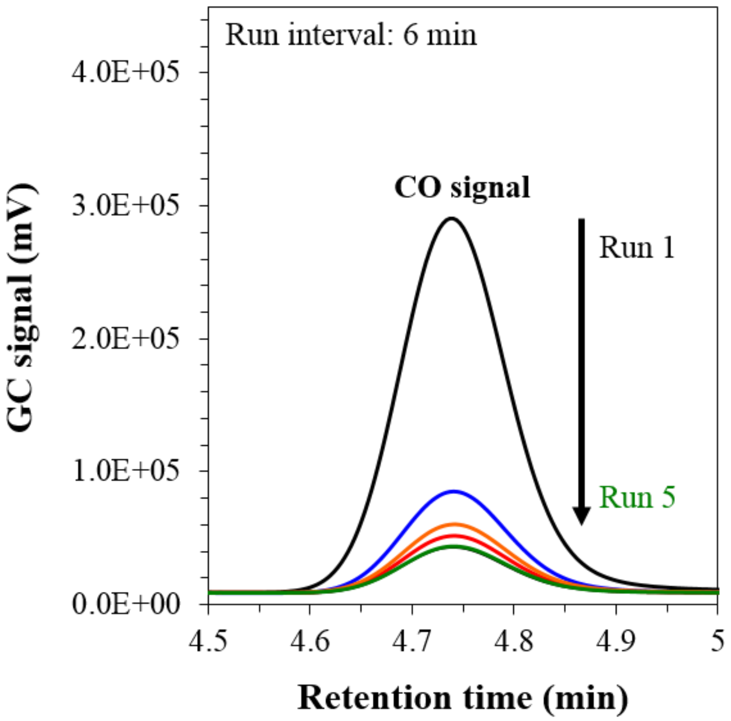

Supplementary Figure 12. Light-induced CO_2 dissociation to CO in absence of H_2. Gas chromatogram of plasmon-induced CO_2 splitting to CO on Al NCs. The CO_2 flow rate was 10 sccm and illumination intensity of 10 W cm^{-2} was utilized. Conversion of CO_2 to CO in the absence of H_2 was found to be viable on plasmonic nanoparticle surface under illumination. However, the conversion efficiency dropped shortly after the reaction started. This can be attributed to poisoning or oxidation of catalyst surface due to formation of oxygen adatoms from C=O band dissociation.

Supplementary Note 1. Calculated local heating on single particle surface.

The maximum temperature increase of the Al@Cu_2O nanostructure was calculated for single particle under pulsed illumination as a function of laser intensity and wavelength (Supplementary Figure 6). The geometry of the structure is identical to the sample in experiments and in electromagnetic simulations. Under the assumption that electron-phonon thermalization occurs much faster than external thermal diffusion (from 100 ps to a few ns) and the time separation between laser pulses, the maximum temporal temperature increase of the particle was calculated separately for Al and Cu_2O by $\Delta T_{max} = \sigma_{abs} F/(V\rho c)$ according to the theory developed in, [1] where σ_{abs} is the absorption cross section of Al (or Cu_2O) in Al@Cu_2O calculated from FDTD electromagnetic modeling, $F = \langle I \rangle / f$ is the laser fluence, $\langle I \rangle$ and f = 80 MHz are the average intensity and pulse repetition rate of laser, respectively. V, ρ and c are the volume, mass density and heat capacity of Al (or Cu_2O).

Supplementary Note 2. General information on Monte-Carlo Simulation.

Because thermal properties in the system derive from the absorption of light, which is not an observable property, calculating light transport properties of the system can give important information about the thermal response. In systems composed of high density of randomly dispersed particles, the Monte-Carlo (MC) method is particularly well-suited for making light transport calculations. Given the nanoparticle density for 5% loading on oxide support and optical cross sections, light absorption and scattering coefficients were calculated. These optical coefficients give the probability per unit propagation that a photon will interact with the collection of nanoparticles, and inverse gives the mean-free path of a photon in the medium. We incorporate these values into a MC simulation by choosing random propagation distances according to the cumulative probability distribution, equation (1):

$$F(x) = 1 - e^{-\mu_e x} \qquad (1)$$

where μe is the extinction coefficient (which is wavelength dependent), and x is the propagation distance. Then whether a photon is absorbed or scattered is chosen randomly according to the albedo. If the photon is scattered, the scattering direction is also chosen randomly. If the photon is absorbed, the position is recorded and the simulation of the next

photon begins. Each photon simulated goes through as many iterations as needed until it is absorbed or leaves the simulation domain. By simulating the fate of many such photons a statistical solution to the light transport properties of the medium can be calculated for each wavelength of light. Of particular interest in this work are the absorption efficiency and absorption density. The latter is directly proportional to the heat-source density, i.e., the source term in the heat equation. Therefore, the temperature increase of the nanoparticle/support matrix should be directly proportional to the heat density.

Supplementary Method 1. TEM characterization.

High-resolution electron micrographs and energy dispersive X-ray spectroscopy (EDX) data were acquired on a JEOL 2100 Field Emission Gun electron microscopes at 200kV (Rice U., U.S.A.). Additional high-contrast (HC) TEM images were collected by a JEOL HC Field Emission Gun electron microscope at 80kV (Rice U., U.S.A.). EDX data were processed using the open source multidimensional data analysis toolbox, HyperSpy. [2]

Supplementary Method 2. XPS characterization.

We performed high resolution XPS analysis on the Al@Cu_2O/γ-Al_2O_3 mixture before and after illumination under reaction condition (Supplementary Figure 4). XPS analysis was performed on a PHI 5500 XPS system equipped with an Al KR monochromator X-ray source operating at a power of 350 W. The pressure in the test chamber was maintained below 1×10^{-9} Torr during the acquisition process.

The prepared Al@Cu_2O/γ-Al_2O_3 mixture was stored under lab atmosphere before performing photocatalysis experiment. In the XPS spectrum before illumination, the lack of satellite peaks at binding energy of 943 eV and 965 eV excludes the existence of Cu (+2). This means that storing the sample in air does not lead to further oxidation of Cu_2O to CuO as Cu(+1) is more stable oxide form of copper under ambient conditions.

For XPS analysis after the illumination under reaction condition, we sealed and separated the reactor from the photocatalysis set up and opened it on-site. The photocatalyst powder was initially loaded on a customized sample holder that allowed convenient and fast transfer of the sample from reaction chamber to XPS chamber followed by fast evacuation of the chamber. A quick (<5 sec) transfer of sample from reaction chamber to XPS chamber minimizes the exposure of the photocatalyst to ambient air atmosphere and thus any possible change in oxidation state of the copper.

The higher resolution XPS analysis at binding energies of copper shows an identical spectrum. It is known that the Cu 2p3/2 spin-orbit peak in metallic copper is narrower than that of Cu(+1). However, we measured similar FWHM of Cu 2p3/2 peak before and after illumination, meaning that the oxidation state of copper should mainly remain unchanged during photocatalytic process. The weak Cu auger peak appears at binding energy of the ~ 570 eV corresponds to the Cu(+1). [3] In addition, we did not observe any change in physical

properties (specifically color) of sample as a result of illumination. Previous studies have reported that polycrystalline Cu_2O (as we observed in our HRTEM images), in particular with a predominant Cu^{+1}-terminated (111) surface, is resistance to photoreduction, while single crystalline Cu_2O is prone to photoreduction. [4] In our Al@Cu_2O nanoparticles, because epitaxial growth of Cu_2O on amorphous aluminum oxide layer surrounding the Al core was not possible, the Cu_2O growth was performed through mild alcoholic reduction without utilizing any capping agent to avoid organic contamination for photocatalytic experiments. As a result, we found the Cu_2O shell very porous and low degree of crystallinity. Overall, although we do not exclude the existence of metallic copper (from reduction of Cu(I) during chemical reaction) in our photocatalysts at percentage below the detection limit of XPS, our combined experimental and simulation results suggest that copper should remain in the form of Cu_2O throughout the photocatalytic reaction.

Supplementary Method 3. Photocatalytic study of rWGS in static mode.

Static mode rWGS experiments were performed on an Al@Cu_2O/γ-Al_2O_3 mixture in a closed chamber configuration (without flowing reactant streams) (Supplementary Figure 11). The reaction chamber was purged with He first and then filled with CO_2 and H_2 at stoichiometric ratio of 1:1 and total pressure of 1 atm. The inlet/outlet valves were closed and the photocatalytic reaction was performed under visible light illumination of $9 Wcm^{-2}$ for 90 min. The valves were then opened and the chamber was purged to toward the gas chromatography system with He gas flowing at 10 sccm into the chamber.

Supplementary References

1. Baffou, G. & Rigneault, H. Femtosecond-pulsed optical heating of gold nanoparticles. *Phys. Rev. B* **84,** 035415 (2011).

2. De la Peña, F. *et al.* hyperspy/hyperspy: HyperSpy 1.1.2, (2017).

3. Wilson, S. S. *et al.* Interface stoichiometry control to improve device voltage and modify band alignment in ZnO/Cu_2O heterojunction solar cells. *Energy Environ. Sci.* **7,** 3606-3610 (2014).

4. Sowers, K. L. & Fillinger, A. Crystal Face Dependence of p-Cu_2O Stability as Photocathode. *J. Electrochem. Soc.* **156,** F80-F85 (2009).
