# Supplementary material for: Plasmon-induced selective carbon dioxide conversion on earth-abundant aluminum-cuprous oxide antenna-reactor nanoparticles
Source: Nat Commun. 2017 Jun 21;8:27. doi: 10.1038/s41467-017-00055-z (PMC5479834; doi:10.1038/s41467-017-00055-z)
Supplement: Supplementary file 2 — TPR [file 41467_2017_55_MOESM2_ESM.pdf]

Reviewers' comments:

Reviewer #1 (Remarks to the Author):

Halas and coworkers describe a plasmon-driven reverse water-gas shift reaction using non-precious Al@Cu_2O nanoparticles as photocatalysts. Compared with traditional thermal-driven reactions, a better selectivity for CO at lower temperature was achieved. The authors further clarified the difference between the contributions of plasmon-induced carrier generation and photothermal heating effect, and investigated the different role of Al core and Cu_2O shell. Overall the concept is intriguing and the results look inspiring. I have several questions before the manuscript can be published.
1) The authors do not show the conversion of CO_2. Based on their data and rough calculation, the conversion is extremely low (<0.1%). The authors should improve the reaction conditions (i.e., decreasing space velocity), otherwise the results are not reliable.
2) On line 207, the authors state: "The overall reaction yield at an intensity of 10 W cm-2 is comparable to the thermal process at temperatures of 400°C. This provides additional evidence that plasmon induced chemical transformations can operate more efficiently and selectively under milder reaction conditions." Considering the low reduction temperature of Cu, are the catalysts chemically the same under the two conditions? This should be supported by XPS data. If not, this comparison does not make sense.
3) Why the plasmon effects of Al on CO_2 conversion were not obvious when without the Cu_2O shell? The role of Cu_2O shell should be proved more clearly on the electron and molecular basis.
4) In figure 4a, the band structure of Cu_2O is misleading. I don't believe the bandgap at air-solid interface is much larger that at solid-solid interface.
5) In figure 4, how are the positive charges transported and compensated? In this regard, will the Al core be oxidized into Al_2O_3? I suggest the authors to test the stability of their catalysts, which they did not mention in the manuscript.
6) The relevant advances on photocatalytic CO_2 conversion should be further discussed and cited (e.g., 10.1002/anie.201602973; 10.1039/C6EE00383D).

Reviewer #2 (Remarks to the Author):

This manuscript demonstrated the fabrication of Cu_2O coated plasmonic Al/Al_2O_3 nanoparticles and present an investigation on their catalytic performance for rWGS via a light driven photocatalytic process. Although they do find some interesting results on selectivity of CO_2 conversion to CO, I find it is very difficult to compare their results to the state-of-the-art performance of similar materials/systems. For instance, the light source used in this report generally have a power density as high as 7 W/cm-2, which is significantly higher than the commonly used standard one (AM 1.5G, 0.1W/cm-2). Nonlinear relationship between the photocatalytic activity and the light intensity has been frequently recognized, exacerbating the uncertainty of the significance of their work. Other points need to be addressed is the material characterization side, for example, the formation of Cu_2O needs to be further confirmed by techniques such as XRD and Raman spectroscopy. Overall, this is a continuous work on Al plasmonic photocatalysis delivering some interesting findings over CO_2 conversion. However, the low quantum efficiency ~ 0.3% (although claimed to be the highest) under very stringent conditions (> 70 sun) enlarges the difficulties in accessing the importance of this work. I would recommend transferring their work to a more specialized journal.

Reviewer #3 (Remarks to the Author):

This manuscript reported a hybrid Al-Cu_2O nanostructure that can achieve a high reactivity and selectivity for CO_2 reduction reaction. The authors focused on differentiating the plasmon-induced carrier-driven mechanism from the phtotothermal heating mechanism. The concerns are that since Al is covered by a self-generated amorphous Al_2O_3 layer, the direct electron transfer from Al to Cu_2O would be limited and the tunneling will have low efficiency. Additionally, it is known that Cu_2O also has absorption in the visible range and itself can function as photocatalysts for CO_2 reduction, making the resonance energy transfer mechanism also possible.

The results demonstrating the selective production of CO is interesting. Is it possible that the selectivity comes from the surface structure of Cu_2O? Experiments or literatures should be included to either exclude or confirm such possibility.

More description of the Monte-Carlo calculations (Fig 3d, 3e and 3f) are needed for general readers to understand. Control experiment for CO_2 reduction using only Cu_2O is also needed. The UV-vis spectra of Al, Cu_2O, and Al/Cu_2O are missing.

In Page 6-7, the authors claimed that the photothermal heating is not responsible for the catalytic activity by comparing the different reaction selectivity of the light-induced and thermal-driven reactions. However, using the spatial temperature mapping to measure the temperature increase under illumination only provide the steady-state temperature that should be much lower than the local temperature on the catalyst surface.

What is the exact oxidation state of Cu_2O during the photoreaction? Recently it has been reported that the oxidation states of Cu_xO can be switched by the plasmon-induced hot carrier injection (i.e., Nature Materials 14, 567–576 (2015)).

REVIEWERS' COMMENTS:

Reviewer #1 (Remarks to the Author):

The authors have finely addressed the referee's comments. The revised manuscript is recommended for publication

Reviewer #2 (Remarks to the Author):

The authors have addressed my concerns and the manuscript can be accepted for publication.

Reviewer #3 (Remarks to the Author):

I am satisfied with the revision and recommend for publication.

Dear Editor:

Thank you for the opportunity to respond to the Reviewers' comments. We address each comment below. We identify where we have edited the manuscript by supplying a version with highlights and line numbers so that each action we have taken to respond can be easily identified.

Thank you for the opportunity to have our manuscript considered for publication in your journal.

Regards, Naomi Halas

Reviewers' comments:

Reviewer #1 (Remarks to the Author):

Halas and coworkers describe a plasmon-driven reverse water-gas shift reaction using non-precious Al@Cu_2O nanoparticles as photocatalysts. Compared with traditional thermal-driven reactions, a better selectivity for CO at lower temperature was achieved. The authors further clarified the difference between the contributions of plasmon-induced carrier generation and photo-thermal heating effect, and investigated the different role of Al core and Cu_2O shell. Overall the concept is intriguing and the results look inspiring. I have several questions before the manuscript can be published.

Response: The authors thank the reviewer for their thorough and positive feedback.

1) The authors do not show the conversion of CO_2. Based on their data and rough calculation, the conversion is extremely low (<0.1%). The authors should improve the reaction conditions (i.e., decreasing space velocity), otherwise the results are not reliable.

Response: We have tested the photocatalytic activity of Al@Cu_2O for the rWGS reaction in a closed-batch reactor (zero space velocity) and have found significant improvement (2X-3X) in the efficiency of CO_2 conversion (Fig. S11). We have chosen to utilize high space velocities throughout the experiment to allow the system to operate in a light-limited regime, rather than a regime limited by the kinetics of surface adsorption (doi:10.1038/nchem.1032; doi:10.1038/nmat3454). Under these conditions, chemical bond activation is limited by the efficiency of hot-carrier generation and injection, which is better suited for studying the photophysical mechanisms of our system.

The primary goal of the present work is to demonstrate how the antenna-reactor concept can be utilized to more efficiently exploit aluminum plasmonics to enhance carrier generation in Cu_2O, and to gain a better understanding of the different ways that carrier-driven and photothermal heating mechanisms contribute in plasmon-driven photocatalysis. Engineering reaction conditions to achieve higher overall conversion efficiencies is the subject of ongoing investigations in our laboratory.

Action: In the revised version of the manuscript, we have discussed the efficiencies of our system in more detail under Fig.2 by comparing our work with the literature on lines 143-155. For additional coherency, we have split Fig. 2 in the original version into two separate figures, now Fig.2 and Fig.3.

2) On line 207, the authors state: "The overall reaction yield at an intensity of 10 W cm^{-2} is comparable to the thermal process at temperatures of 400°C. This provides additional evidence that plasmon induced chemical transformations can operate more efficiently and selectively under milder reaction conditions." Considering the low reduction temperature of Cu, are the catalysts chemically the same under the two conditions? This should be supported by XPS data. If not, this comparison does not make sense.

Response: Comparing the reaction rate under illumination with the corresponding thermal process at elevated temperatures is valid, even if the composition of the material changes at elevated temperatures, because it highlights one of the main advantages of light-driven processes over traditional thermal catalysis. That is, to drive chemical transformations at low temperatures using photogenerated carriers not only can have positive impacts on overall energy consumption, but may also increase the lifetime of the catalyst. The instability of metals and metal oxides at high operating temperatures for chemical conversion has been frequently reported in the literature. Yet, high temperature is often required to activate the catalyst to initiate chemical conversion in thermally driven processes. Lower operating temperatures have been shown to significantly increase the lifetime of catalysts (doi:10.1038/nchem.1032).

Action: We have replaced that statement with a clarified version in the main text. Please review lines 214 to 221 of the revised manuscript for our response.

3) Why the plasmon effects of Al on CO_2 conversion were not obvious when without the Cu_2O shell? The role of Cu_2O shell should be proved more clearly on the electron and molecular basis.

Response: The wavelength-dependent CO formation on uncoated Al is compared with Al@Cu_2O in Fig.4a of the revised manuscript. The reason that the contribution from the LSPR of Al is not as efficient as in Al@Cu_2O can be explained based on the contribution of the plasmon to enhanced-carrier generation and differences in surface catalytic properties. For pristine Al NCs, only non-radiative plasmon decay contributes to carrier generation for C=O bond activation. For the 100 nm Al NCs in this study, the major branching pathway for plasmon decay is through the radiative channel, which cannot be utilized to convert CO_2.

The role of the Cu_2O shell is two-fold: First, to accept energy transfer from radiative plasmon decay, which can induce interband or subband optical transitions, subsequently generating additional carriers for chemical bond activation. This is supported by our electromagnetic simulations (Fig.4b). Second, from a molecular perspective, the surface chemistry of Cu_2O can enhance CO_2 adsorption and reduce the energy barrier for carrier injection into adsorbed CO_2. We have provided a more detailed discussion about the molecular role of Cu_2O surface chemistry in the revised version of manuscript.

Action: We have provided additional discussion based on the reviewer's comments on lines 329-350 and 378-405 for the electron and molecular roles of Cu_2O, respectively.

4) In figure 4a, the band structure of Cu_2O is misleading. I don't believe the bandgap at air-solid interface is much larger that at solid-solid interface.

Response: The authors agree, and appreciate the reviewer for catching this mistake.

Action: An updated figure has been embedded in the revised version of the manuscript (line 351). The new figure better describes the structure and mechanism of plasmon-induced carrier-assisted rWGS on Al@Cu_2O.

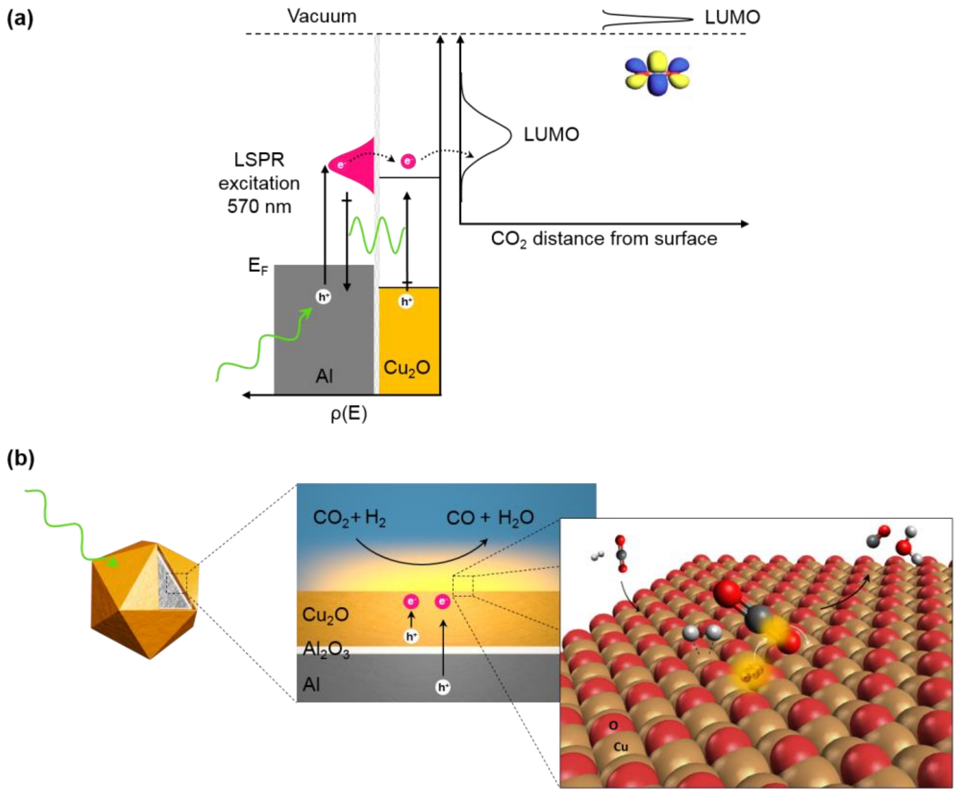

Figure 5. Structure and mechanism of plasmon-induced carrier-assisted rWGS on Al@Cu_2O. a) Energy band diagram of Al@Cu_2O for plasmon-mediated carrier generation for injection into unoccupied state of CO_2 for C-O bond activation. b) Schematic of plasmon-induced carrier-driven rWGS on Al@Cu_2O.

5) In figure 4, how are the positive charges transported and compensated? In this regard, will the Al core be oxidized into Al_2O_3? I suggest the authors to test the stability of their catalysts, which they did not mention in the manuscript.

Response: Charge-carrier driven transformations on plasmonic nanostructures is a neutral process in which the energy of carriers is deposited into specific vibrational modes of the adsorbates to promote the molecule to higher vibrational states, before returning to the nanostructure surface to recombine (doi:10.1038/nchem.1032, doi:10.1038/nmat3454). Because the carriers eventually recombine, the plasmonic antenna (Al) remains intact and the positive charges do not oxide the core. The excellent stability of Al during plasmon-driven photocatalysis has been demonstrated in our recent publication (DOI: 10.1021/acs.nanolett.6b03582). In the present study, the Al@Cu_2O particles showed very good stability over several days of continuous experimentation as verified by XPS analysis before and after illumination in Fig S4.

Action: We have explained the mechanism of plasmon-induced carrier-driven chemical transformation in more detail on lines 339-350, and Fig. 5 (line 351) in the revised manuscript illustrates the mechanism of plasmon-induced carrier-assisted rWGS on Al@Cu_2O in more detail.

6) The relevant advances on photocatalytic CO2 conversion should be further discussed and cited (e.g., 10.1002/anie.201602973; 10.1039/C6EE00383D).

Response: Thanks for your suggestion.

Action: We have cited the relevant references in their proper places (line 325 and 328).

Reviewer #2 (Remarks to the Author):

This manuscript demonstrated the fabrication of Cu_2O coated plasmonic Al/Al_2O_3 nanoparticles and present an investigation on their catalytic performance for rWGS via a light driven photocatalytic process. Although they do find some interesting results on selectivity of CO_2 conversion to CO, I find it is very difficult to compare their results to the state-of-the-art performance of similar materials/systems. For instance, the light source used in this report generally have a power density as high as 7 W/cm^{-2}, which is significantly higher than the commonly used standard one (AM 1.5G, 0.1W/cm^{-2}).
Other points need to be addressed is the material characterization side, for example, the formation of Cu_2O needs to be further confirmed by techniques such as XRD and Raman spectroscopy. Overall, this is a continuous work on Al plasmonic photocatalysis delivering some interesting findings over CO_2 conversion. However, the low quantum efficiency ~ 0.3% (although claimed to be the highest) under very stringent conditions (> 70 sun) enlarges the difficulties in accessing the importance of this work. I would recommend transferring their work to a more specialized journal.

Thank you for your feedback.

Response: Improving quantum yield is the Holy Grail in all photocatalytic processes, and in particular for CO_2 conversion. However, the present manuscript does not aim to emphasize CO_2 conversion efficiency, but rather demonstrate the first example of hot-

carrier driven highly selective rWGS using a plasmonic system that entirely relies on non-precious, earth-abundant materials. To date, all reported plasmonic systems for photocatalytic CO_2 conversion (and the majority of other reactions) utilize Ag and Au. While they are interesting for fundamental studies, their practical applications are limited. Here, we take a first step by overcoming the challenges related to the cost, abundance, and hence the feasibility of implementing plasmonic systems for light-driven photocatalysis of important chemical transformations. In addition, for the first time, we have addressed the ambiguities related to collective photon absorption in plasmonic photocatalysis systems and have clearly differentiated the contribution of photothermal heating and hot-carrier driven mechanisms in plasmon-enhanced catalysis. These are important points that we ask the reviewer to keep in mind while reevaluating this work for Nature Communications.

We agree that it is very difficult to compare our results with the literature. This is not because of our experimental conditions, but rather the significant diversity of utilized materials and reported experimental conditions for photocatalytic CO_2 conversion. Even though there are dozens of reports using AM 1.5 (0.1 W cm^{-2}) illumination, in the majority of cases the photocatalysts is only active under applied electrochemical bias, or in the presence of sacrificial organics. We acknowledge the high light intensity used in our study, although we do not utilize precious materials, sacrificial organics or electrochemical bias. Our measured CO formation rate of 360 µmole hr^{-1} on Al@Cu_2O is on par with and even higher than many previous reports of photocatalytic CO_2 conversion (for details please see table I and II of DOI: 10.1021/acscatal.6b02089).

We believe it is important to compare the apparent quantum efficiency of our system with other plasmonic systems that have reported photocatalytic CO_2 conversion. Cronin and co-workers reported a quantum efficiency of 0.002% for CO_2 conversion on Au/TiO_2 (DOI: 10.1021/cs2001434); an apparent quantum efficiency of 0.03% was reported for rWGS reaction on Au/ZnO by C. Wang et, al (DOI: 10.1039/C3NR02001K). A very recent example is photocatalytic CO_2 conversion to CO on a Ag nanocube@Re-MOF photocatalyst (DOI: 10.1021/jacs.6b11027). The authors reported a very low turnover rate of about 0.0108 (hr^{-1}) for CO formation in an organic solvent, while utilizing a sacrificial reagent, and a complex plasmonic system that entirely relies on the precious metals Ag and Re. Our measured quantum efficiency of 0.3%, all while utilizing a non-precious, earth abundant Al@Cu_2O photocatalyst is on par with, or higher than, previous reports on plasmonic CO_2 conversion on Au and Ag nanostructures.

Action: In the revised version of manuscript, we have discussed and compared our efficiencies with literature reports on photocatalytic CO_2 conversion as well as similar plasmonic systems for CO_2 conversion (Figure 2 and line 143-155 of revised version). For additional coherency, we have split Fig. 2 in original version into two separate figures, now Fig.2 and Fig.3.

-Nonlinear relationship between the photocatalytic activity and the light intensity has been frequently recognized, exacerbating the uncertainty of the significance of their work.

Response: It was previously demonstrated (doi:10.1038/nmat3454) that the rate of plasmon-driven photocatalytic processes exhibit a linear dependence on light intensity with a transition from linear to supralinear at higher intensities. This change follows a power-law dependence at higher intensities with rates proportional to intensity^n where n>1. The light intensity at which the transition occurs depends on plasmonic material, particle geometry, the chemical reaction, and temperature amongst other conditions. For example, a similar transition has been reported on extended metal surfaces but at significantly higher (>10^7 times) light intensities than for plasmonic nanoparticles (https://doi.org/10.1103/PhysRevLett.77.1338). Also, it was previously reported that the transition from a linear to a super-linear regime is less pronounced under conditions where both light and thermal energy are applied (doi:10.1038/nmat3454).

In our case, we measured the reaction rate and quantum efficiency as a function of illumination intensity on Cu_2O, Al and Al@Cu_2O (revised Fig. 2). For the case of pristine Al nanoparticles, a transition from linear to supralinear was observed at high light intensities, as expected for a purely plasmonic nanoparticle. However, for Al@Cu_2O the reaction rate dependence on illumination intensity remains more and less linear for the range of illumination intensities tested, while the quantum efficiency starts to level off at higher illumination intensities. The case of Al@Cu_2O is more complex due to two possibilities for carrier generation. Nonradiative plasmon decay can lead to carriers in the Al core (similar to pristine Al case) and radiative plasmon decay can generate charge carriers near the Cu_2O semiconductor surface (which does not exist when uncoated Al NCs were used). For plasmonic nanoparticles, the photocatalytic rate is equal to intensity^n where n>1. Semiconductors exhibit photocatalytic reaction rates equal to intensity^n where n<1. Therefore, increased irradiation intensities on semiconductors does not improve quantum efficiency as verified by pure Cu_2O in revised version of Fig. 2b (DOI: 10.1021/jp0530451; DOI: 10.1021/ja200086g). Because of complexities of carrier generation in our metal/semiconductor hybrid, Al@Cu_2O, we do not expect to see an identical dependence on light intensity in both cases. Additionally, the temperature increase in our system due to photothermal heating results in less pronounced nonlinear effects also contributing to the intensity dependent photocatalytic activity.

Action: We have provided more clarification on differentiating the plasmon-induced carrier-driven mechanism and photothermal heating in our revised version (Fig.2, Fig. 3, Fig. 4, Fig. S6-S7, lines 177-221, 243-246, and 301-3011). iii) A more detailed discussion on the mechanism of plasmon-induced carrier-assisted rWGS on Al@Cu_2O has been included (Fig. 5, and lines 318-378, and 406-420).

Other points need to be addressed is the material characterization side, for example, the formation of Cu_2O needs to be further confirmed by techniques such as XRD and Raman spectroscopy.

Response: XRD was performed on Al@Cu_2O nanoparticles, but because of low penetration depth due to a very thin Cu_2O shell layer, the low crystallinity of the Cu_2O grown on the amorphous aluminum oxide layer surrounding the Al core, and interference

from Al_2O_3 and Al in XRD spectrum, we were not able to resolve features corresponding to Cu_2O. Similarly, we did not detect Cu_2O features in Raman spectroscopy due to very low peak intensities. However, we have provided solid evidence to confirm Cu_2O formation, including a combination of HRTEM (Fig. 1), EDX (Fig. 1) and XPS (Fig. S4) analysis in addition to our electromagnetic simulation results.

Reviewer #3 (Remarks to the Author):

This manuscript reported a hybrid Al-Cu_2O nanostructure that can achieve a high reactivity and selectivity for CO_2 reduction reaction. The authors focused on differentiating the plasmon-induced carrier-driven mechanism from the photothermal heating mechanism.

Response: The authors thank the reviewer for their thorough review.

1. The concerns are that since Al is covered by a self-generated amorphous Al_2O_3 layer, the direct electron transfer from Al to Cu_2O would be limited and the tunneling will have a low efficiency.

Response: This is a very reasonable concern. Generally, a thin layer of oxide layer can significantly reduce the tunneling probability. Despite the 2-4 nm self-limiting amorphous Al_2O_3 layer surrounding the Al core, tunneling of the hot carrier to the oxide surface is still viable (although at low efficiencies) due to a high-density of defect states in the amorphous Al_2O_3 layer. The direct dissociation of adsorbed CO_2 on pristine Al NCs under visible light illumination at ambient conditions provides evidence for this hot-carrier tunneling mechanism (Fig. S12). The formation of CO in absence of H_2 justifies the hypothesis of hot-carrier transfer into unoccupied orbitals in CO_2 for C-O bond dissociation. Photodissociation of non-adsorbed CO_2 would not be possible, which requires incident photons at vacuum ultraviolet energies (>10 eV) (DOI: 10.1039/C3CP53250J).

We would like to clarify that we believe the more important mechanism here is the energy transfer via radiative decay of the Al LSPR to Cu_2O for enhanced-carrier generation. For the 100 nm Al NCs used in this study, the major plasmon decay channel is through the radiative mechanism and thus carrier generation from nonradiative decay of the plasmon in the Al core is not very efficient. Accordingly, the rate of CO formation in pristine Al NCs is significantly lower that of Al@Cu_2O.

Action: We discussed this point in more detail within lines 371-377 of the revised version of manuscript.

2. Additionally, it is known that Cu_2O also has absorption in the visible range and itself can function as photocatalysts for CO_2 reduction, making the resonance energy transfer mechanism also possible.

Response: The authors agree. The energy transfer mechanism is one of the main points that we emphasized in the original manuscript (lines 378-405).

Action: We have included a new paragraph in the revised version of manuscript describing the resonance energy transfer mechanism in more detail (406-420).

3. The results demonstrating the selective production of CO is interesting. Is it possible that the selectivity comes from the surface structure of Cu_2O? Experiments or literatures should be included to either exclude or confirm such possibility.

Response: The enhanced selectivity for CO formation in our study is the results of plasmon-mediated process rather than surface structure of Cu_2O alone. The selectivity observed during light-induced processes on Al@Cu_2O can be explained based on plasmon-induced selective C=O bond activation and desorption of CO* intermediate from reactive surface sites. The net energy transfer from surface plasmon to intermediates on the surface can induce a nonthermal desorption of CO* intermediates (Physical Review Letters 2007, 99(22): 225501). Plasmon-induced selective CO formation was found to be independent of surface chemistry as it was observed for light-induced rWGS on both pristine Al and Al@Cu_2O photocatalysts. In addition, varying the ratio of H_2 to CO_2 in steam gas had negligible effect on product selectivity for light-induced rWGS, which exhibited selectivity towards CO of over 99.3% (Fig. S10). Previous work conducted on the rWGS on oxide supported Au NPs and found selective CO formation (DOI: 10.1039/c4cy01183j). A very recent example of plasmon-induced conversion of CO_2 to CO has been reported over Ag nanocube@Re-MOF photocatalyst (DOI: 10.1021/jacs.6b11027). In our laboratory, we also performed rWGS on Ag nanoparticles under similar experimental condition and similarly found selective CO formation under illumination (data not included in the present manuscript).

Action: We have provided more detailed discussion on plasmon-enhanced selectivity of CO_2 conversion and cited the relevant reference in lines 356-370 of revised version of manuscript.

4. More description of the Monte-Carlo calculations (Fig 3d, 3e and 3f) are needed for general readers to understand.

Action: We have included more description of the Monte-Carlo simulation to the supplementary information (section S1) of the revised version of manuscript.

5. Control experiment for CO_2 reduction using only Cu_2O is also needed.

Response: We have prepared Cu_2O through an alcoholic reduction method similar to the technique used to grow a Cu_2O layer around Al. Under experimental condition similar to that of Al@Cu_2O, we have observed negligible photocatalytic activity for rWGS using these Cu_2O particles.

Action: We performed control experiment for CO_2 conversion using Cu_2O. The data are included in revised version of Fig. 2.

6. The UV-vis spectra of Al, Cu_2O, and Al/Cu_2O are missing.

Action: The corresponding UV/Vis spectra have been included under supplementary information (Fig. S2).

7. In Page 6-7, the authors claimed that the photothermal heating is not responsible for the catalytic activity by comparing the different reaction selectivity of the light-induced and thermal-driven reactions. However, using the spatial temperature mapping to measure the temperature increase under illumination only provide the steady-state temperature that should be much lower than the local temperature on the catalyst surface.

Response: The local temperature increase should not be much higher than the steady-state surface temperature measured by high-resolution spatial mapping of the catalysts surface because the system reaches equilibrium shortly after illumination. In equilibrium, the magnitude of heat generation and accumulation is equal to the magnitude of heat diffusion and heat transfer to the environment and stainless steel stage. Also, the multiple scattering event by randomly dispersed particles under illumination volume increase the magnitude of heat diffusion as can be seen in Fig.3a, and thus prevent significant heat accumulation in a small area under illumination.

Recent steady-state measurements of the local surface temperature using *in situ* Raman spectroscopy were recently demonstrated during photocatalytic rWGS on Au NPs in a ZnO matrix (DOI: 10.1039/c3nr02001k). The authors reported a surface temperature of about 30°C for illumination intensities of 12 W cm^{-2} (higher than our maximum laser intensity of about 10 W cm^{-2}) at plasmon resonance of Au (532 nm). We also calculated the maximum local temperature on the surface of the single Al@Cu_2O was calculated as a function of illumination intensity and wavelength (Fig. S6 was added to revised version of the manuscript), which resulted in negligible temperature increase under illumination conditions. However, our experimental study of high-resolution spatial and temporal mapping of temperature variations during irradiation (Fig. 3a,b and Fig. S7) showed that temperature increases near the illuminated surface at our maximum visible light intensity is significantly higher than previously reported and our calculated local temperature increase for single particle surface. The difference can be attributed to the collective heating effect mediated by multiple light scattering and absorption events by randomly dispersed particles under illumination volume (DOI: 10.1021/nl5016975). If the local temperature is significantly higher than what was measured steady-state surface temperature, then we would expect a photothermal-driven rWGS under illumination and a loss of CO selectivity. However, despite temperature increases due to plasmonic photothermal heating, we provided definitive evidences in Fig. 3 and Fig. 4 that photocatalytic rWGS on Al@Cu_2O is driven through plasmon-induced carrier-assisted mechanism.

Action: We have included calculated local temperature of the Al@Cu_2O for a single particle surface (Fig. S6), and provided corresponding detailed discussion and relevant references within lines 177-199. Also, for more clarification on differentiating the plasmon-induced carrier-driven mechanism with photothermal effects, we rearranged the

Fig. 2 in original manuscript into two separate figures (Fig. 2 and Fig.3). Fig.3 now mainly focuses on describing our results on differentiating these two effects. This is followed by more evidence in Figure 4 to further corroborate plasmon-induced carrier-assisted rWGS under illumination. Accordingly, we have made several changes within the main text and have provided more detailed discussion within lines 172-242 and 301-311.

8. What is the exact oxidation state of Cu_2O during the photoreaction? Recently it has been reported that the oxidation states of Cu_xO can be switched by the plasmon-induced hot carrier injection (i.e., Nature Materials 14, 567–576 (2015)).

Response: Thanks for your feedback. This is very good question and the short answer is that the oxidation state of Cu_2O during photoreaction remains as Cu_2O.

In the work by Linic and co-workers (the original published in Science, DOI: 10.1126/science.1231631), the authors demonstrated that hot-carrier injection from LSPR excitation in Cu, it is possible to reduce the outer Cu_2O shell that was formed during propylene epoxidation on Cu NPs. The magnitude of reduction of Cu_xO depends on factors, such as degree of crystallinity, efficiency of hot-carrier injection, reduction temperature. In their work, Cu_2O was formed from the oxidation of surface Cu atoms with high crystallinity as indicated by XRD, and an epitaxial allowed for efficient carrier injection at relatively higher operating temperatures of about 473 K.

The Cu_2O was grown around Al core was highly polycrystalline as epitaxial growth of Cu_2O on amorphous aluminum oxide layer surrounding the Al core was not possible. It has been reported that polycrystalline oxides are more resistant to photoreduction than single crystalline oxides (doi: 10.1149/1.3089290). Consistently, we did not notice any change in physical property (color) of sample as a under illumination. The XPS spectrum of Al@Cu_2O nanoparticles (see below) before and after illumination under experimental condition is almost identical with similar FWHM measured for Cu $2p_{3/2}$ (also see Fig. S4 for more information).

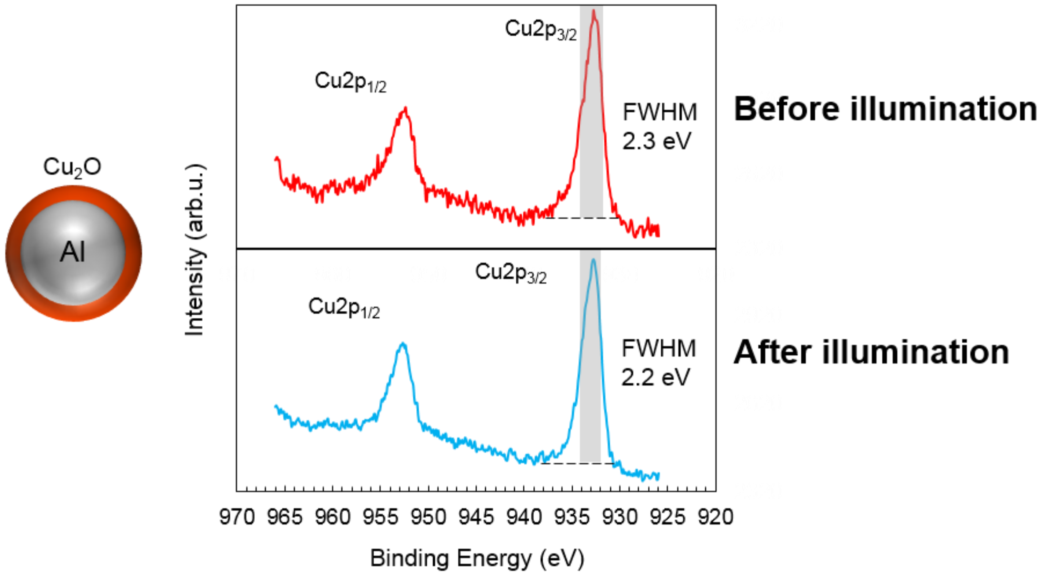

Action: We have provided a more detailed discussion of XPS analysis in the revised manuscript (lines 108-112 and Fig. S4)
